# Unraveling the microbial processes of black band disease in corals through integrated genomics

**DOI:** 10.1038/srep40455

**Published:** 2017-01-17

**Authors:** Yui Sato, Edmund Y. S. Ling, Dmitrij Turaev, Patrick Laffy, Karen D. Weynberg, Thomas Rattei, Bette L. Willis, David G. Bourne

**Affiliations:** 1 Australian Institute of Marine Science, PMB 3, Townsville MC, Townsville 4810, Australia; 2 Global Change Institute, School of Agriculture & Food Sciences, The University of Queensland, Brisbane 4072, Australia; 3 Department of Computational Systems Biology, University of Vienna, Althanstrasse 14, 1090 Vienna, Austria; 4 ARC Centre of Excellence for Coral Reef Studies, James Cook University, Townsville 4811, Australia; 5 College of Science and Engineering, James Cook University, Townsville 4811, Australia

## Abstract

Coral disease outbreaks contribute to the ongoing degradation of reef ecosystems, however, microbial mechanisms underlying the onset and progression of most coral diseases are poorly understood. Black band disease (BBD) manifests as a cyanobacterial-dominated microbial mat that destroys coral tissues as it rapidly spreads over coral colonies. To elucidate BBD pathogenesis, we apply a comparative metagenomic and metatranscriptomic approach to identify taxonomic and functional changes within microbial lesions during *in-situ* development of BBD from a comparatively benign stage termed cyanobacterial patches. Results suggest that photosynthetic CO_2_-fixation in Cyanobacteria substantially enhances productivity of organic matter within the lesion during disease development. Photosynthates appear to subsequently promote sulfide-production by Deltaproteobacteria, facilitating the major virulence factor of BBD. Interestingly, our metagenome-enabled transcriptomic analysis reveals that BBD-associated cyanobacteria have a putative mechanism that enables them to adapt to higher levels of hydrogen sulfide within lesions, underpinning the pivotal roles of the dominant cyanobacterium within the polymicrobial lesions during the onset of BBD. The current study presents sequence-based evidence derived from whole microbial communities that unravel the mechanism of development and progression of BBD.

Coral reefs are currently facing global declines, with coastal development, over-fishing, anthropogenic pollution, global warming, ocean acidification and intense storm activities contributing, either in isolation or combination, to these declines^1^,^2^,^3^. Increasingly, coral diseases have been recognized as another major contributor to on-going reef degradation^4^. For example, approximately 7% of long-term declines in coral cover on the Great Barrier Reef (GBR) have been attributed to coral disease^5^, and a Belizean reef in the Caribbean has undergone a community phase shift owing to a large-scale disease outbreak in the late 1980s^6^. Impacts and frequency of coral disease outbreaks are expected to further increase when corals are affected by accumulating environmental stressors^7^. However, the etiologies of most coral diseases are yet to be elucidated, hindering the development of effective management plans against disease outbreaks.

Here, we apply comparative metagenomic and metatranscriptomic approaches to elucidate the underlying microbial mechanisms responsible for the development and progression of black band disease (BBD) in corals. BBD is a virulent disease that manifests as a microbial mat (lesion), which migrates across coral colonies, killing the underlying coral tissue rapidly^8^,^9^,^10^. BBD represents a polymicrobial disease since interactions of different microbial groups within the lesion appear to be essential to the etiology. The BBD lesion is dominated by cyanobacteria, though also includes an array of other microorganisms, such as sulfur-cycling bacteria, heterotrophic bacteria, Archaea, Fungi and other Eukaryotes^10^. The complexity of the BBD lesion community has challenged the mechanistic understanding of the underlying etiology leading to the onset of the disease.

A recent field- and molecular-based study reported that some cases (at least 19% of directly confirmed cases) of BBD on an inshore GBR reef are derived from comparatively benign lesions termed ‘cyanobacterial patches’ (CP; Fig. 1)^11^. Although early developmental stages of BBD may also manifest as lesions other than CP, successional changes in microbial communities during the development of BBD from CP have been extensively investigated and documented as a model study system of BBD pathogenesis^10^. Key changes during the development of BBD from CP include (1) an increase in the overall virulence of the lesion, as measured by progression rates on coral tissue^11^, (2) a shift in the dominant cyanobacterium to a phylogenetically distinct species^11^, (3) increased relative proportions of sulfide-reducing bacteria (SRB) and decreased relative abundance of sulfide-oxidizing bacteria (SOB)^12^,^13^, (4) a shift in the archaeal community composition to a community dominated by a novel archaeal species^14^, and (5) the formation of anoxic and sulfide-rich microenvironmental conditions within BBD lesions, with diurnal dynamics, that are correlated to the overall virulence^15^ (see Fig. 1). Although what triggers CP remains to be investigated, the discovery of these changes during the transition from CP to BBD represented an important step towards a mechanistic understanding of BBD pathogenesis. Findings from the CP-BBD system have been synthesized into a conceptual model to illustrate the development of BBD pathogenicity^10^, however a detailed understanding of microbial drivers contributing to the development of virulence have not been achieved due to the lack of investigations into the functions of microbial members and their interactions at a whole lesion community scale. In the present study, microbial lesions associated with the precursor CP stage and the more virulent BBD stage were collected during the onset of BBD from individually-monitored corals *in situ*. The microbial community structure and functional properties were directly compared between CP and BBD to identify the mechanisms playing central roles in the development of increased virulence specific to the BBD lesion.

## Materials and Methods

### Study site and collection of specimens

Assemblages of the coral *Montipora* spp. were monitored along the east coast of Pelorus Island, located in the central inshore GBR (18°33′S, 146°30′E). Replicate colonies of *Montipora hispida* infected with CP were haphazardly selected and individually marked underwater, and development of the characteristic signs of BBD (*i.e.* a darkly pigmented microbial mat formed at the interface between apparently healthy coral tissue and exposed white skeleton) was followed *in situ* between 2009 October and November at weekly intervals. More information on environment conditions and seasonal dynamics of CP and BBD at the study site can be found in the previous studies by Sato *et al*.^11^,^16^. Microbial mat specimens were obtained from three coral disease lesions that were monitored as they underwent transitions from the precursor CP stage to fully developed BBD lesions in two to three weeks. To minimize impacts of sampling on the development of microbial lesions, only a small portion of the microbial mat, approximately 5 mm in diameter and 2 mm in depth, including the top surface of the microbial lesion and attached underlying skeleton, was collected from the progressing front of the lesion^11^. Specimens were collected during the daylight (11:00–13:00) in duplicate for extraction of DNA and RNA at each time point (Suppl. Table 1), using a separate sterilized stainless steel chisel for each lesion to avoid cross-contamination. Materials collected were placed in 1.5 ml sterile nuclease-free plastic tubes underwater. Lesion samples for DNA-sequencing were kept on ice and transported to the laboratory. Within 4 hours of sampling, seawater was removed from tubes and replaced with 100% ethanol, and the specimens were stored at −20 °C prior to DNA extraction^11^. For RNA-targeted specimens, seawater contained in the 1.5 ml sample tubes was removed immediately after sampling and replaced with RNAlater (Ambion, Austin, TX). Specimens were kept on ice while being transported to the laboratory and stored at −20 °C prior to RNA isolation. The morphology of cyanobacterial filaments within a small unpreserved subsample of each lesion was observed under a phase contrast microscope (BX41TF, Olympus Corporation, Tokyo, Japan; magnification of 400 times) to identify the developmental stage of BBD in the transition from CP^11^. Developmental stages were further confirmed by profiling cyanobacterial 16 S r RNA-coding genes within DNA samples, using terminal restriction fragment length polymorphism (T-RFLP), as detailed previously in Sato *et al*.^11^.

### Nucleic acid extraction

Total DNA was extracted from ethanol-preserved lesion specimens with the PowerPlant DNA Isolation Kit (MO BIO Laboratories, Carlsbad, CA), as described by the manufacturer, but with a minor modification: following the initial preparation, a 30 sec bead-beating cycle was performed twice with a Mini-Beadbeater-96 (Biospec Products, Bartleville, OK) with a 30 sec interval^11^. Quality of extracted DNA was verified on a 1% agarose gel stained with ethidium bromide and quantified with a NanoDrop2000 Spectrophotometer (Thermo Fisher Scientific, Waltham, MA). Total RNA was extracted from the RNAlater-preserved specimens using the RiboPure Bacteria Kit (Ambion) by following the manufacturer’s instructions. In addition to a DNA-removal step included in the RiboPure kit protocol, total RNA extracted was treated with the TURBO DNA-free kit (Ambion) to completely remove contaminating DNA and cleaned with the RNeasy MinElute Cleanup kit (Qiagen, Hilden, Germany). The quantity and quality of total RNA were checked with the RNA 6000 Pico Kit (Agilent, Santa Clara, CA, USA) on a 2100 Bioanalyzer (Agilent). Equal amounts of DNA or RNA extracted from three individual lesions were pooled within the same disease stage (CP or BBD; see Suppl. Table 1), resulting in a total amount of 2 μg for DNA and 200 ng for RNA. Pooling of DNA and RNA samples within stages was necessary to obtain sufficient quantities of nucleic acids for metagenomic and metatranscriptomic sequencing, as sample biomasses were minimized at each time point in the sampling scheme to best persevere a time-series of *in-situ* pathogenesis for each coral. Analyses in this study, therefore, have focused on comparing the average characteristics of CP and BBD-associated microbial lesions, while enabling identification of significant differences via statistical analyses of high-throughput sequencing data (see ‘Statistical analyses’ below)^17^.

### Microbial messenger-RNA enrichment

Enrichment of microbial messenger RNA (mRNA) from total RNA was performed based on sample-tailored rRNA-subtraction developed by Stewart *et al*.^18^, supplemented with an additional step to remove poly(A)-tailed eukaryotic mRNA^19^ (see Supplementary document for the detailed protocol). In brief, total RNA was hybridized with biotinylated probes targeting small and large subunits of bacterial, archaeal and eukaryotic rRNA (total six probe types). These probes were generated with PCR-amplification of corresponding DNA samples obtained from different disease developmental stages (CP and BBD; *i.e.* a total of 12 types of rRNA probes were synthesized). Hybridized rRNA was then removed from total RNA with streptavidin-coated magnet beads. To subtract poly(A)-tailed mRNA from the remaining RNA, oligo d(T)-coated magnetic beads were added subsequently and hybridized beads were removed. Enrichment of microbial mRNA was verified on a 2100 Bioanalyzer (Agilent); ensuring a substantial decline of signature peaks for small and large subunit rRNA in RNA size distribution profiles. Microbial mRNA-enriched RNA was linearly amplified using the MessageAmp II Bacteria kit (Ambion), and double-stranded cDNA was synthesized for each CP and BBD sample.

### Metagenomic and metatranscriptomic sequencing

For metagenomic sequencing, paired-end libraries with average insert sizes of approximately 400 nt were generated from CP and BBD-derived DNA and sequenced by the Australian Genome Research Facility (Brisbane) using the Illumina GAIIx sequencing system (Illumina, San Diego, CA), yielding 101 nt paired-end sequences. For metatranscriptomic sequencing, cDNA was cleaned, size-selected (approximately between 120 and 1180 base pairs) and sequenced at the Ramaciotti Centre (Sydney) using Illumina HiSeq2000 technology (Illumina), yielding 101 nt paired-end sequences. Sequence analyses and statistical comparisons were computed using the software described below with default parameters unless otherwise specified.

### Metagenomic analyses

The quality of paired-end Illumina sequences was first checked with FastQC (http://www.bioinformatics.babraham.ac.uk/projects/fastqc/). Metagenomic sequences were clustered at 99% sequence identity using CD-HIT^20^ for each individual CP and BBD library, and representative sequences were identified. For taxonomic and functional assignment of the metagenomes, representative sequences were checked against the NCBI-nr protein database (http://ncbi.nlm.nih.gov/) and a universally conserved proteins (UCP) database (a collection of publicly available sequences based on 31 Clusters of Orthologous Groups that are shared by 99% of Bacteria, Archaea, and Eukaryotes, e.g. sequences coding ribosomal proteins, rRNA-binding proteins and transfer RNA-synthetases)^21^ using RAPSearch2^22^ and BLASTx^23^, respectively, with a threshold of E-values ≤ 10^−5^. Sequence search results against NCBI-nr and UCP databases were parsed into hierarchal classifications according to the NCBI taxonomy (http://www.ncbi.nlm.nih.gov/Taxonomy/) and the SEED functional subsystems^24^ using MEGAN5 software (paired-end sequence reading mode, use magnitude = on, maxMatches = 250; minSupport = 10; minScore = 50.0; maxExpected = 1E-05; topPercent = 10.0; minComplexity = off)^25^.

### Assembly and binning of metagenomes

Metagenome sequences from the CP and BBD individual datasets and the combined dataset were separately assembled using Ray assembler (kmerLength = 31)^26^. Assembled contigs were initially filtered with a minimal length of 1,000-bases and a minimal mean coverage of 3 to remove most potential artefacts. Contigs containing taxonomic marker information were identified using Amphora^27^ and mmgenome (https://github.com/MadsAlbertsen/mmgenome). Groups of contigs longer than 5,000 nt sharing similar tetramer compositions were also identified using CONCOCT (maximum cluster number = 100)^28^. Contigs longer than 5,000 nt were then manually grouped into ‘prebins’ according to consistent information based on available evidence above (*i.e.* CONCOCT-derived grouping, Amphora- and mmgenome-derived taxonomic information) with some manual curation based on differential coverages within CP and BBD libraries, and percentage GC-content. Depending on differential coverage, prebins were originated from either individual CP- or BBD-assembly, or the combined assembly. If contigs in a prebin candidate had substantially different coverages between CP- and BBD-libraries (*i.e.* Cam, Cya1-2, Cty1-3), the individual CP- or BBD-assembly with higher coverage was selected to increase the quality of resulting sequence bins. The CP-BBD combined assembly was selected for other prebins (*i.e.* Alpha1-5, Alt1-4, Fla1-2, Oce). Contigs less than 5,000-bases were subsequently recruited into the resulting prebins to identify final genomic bins using Phymm^29^, with a combined reference dataset consisting of the default Phymm-genomes and all prebin-sequences. Quality of sequence binning was evaluated with CheckM^30^. Gene prediction and functional annotation of predicted protein sequences within each bin were computed using ConsPred (https://sourceforge.net/projects/conspred/) with UniProt, SwissProt and TrEMBL^31^,^32^ as reference databases and consistently annotated functions were identified for each of the predicted gene regions.

### Metatranscriptomic analyses

The quality of metatranscriptomic sequences was first checked with FastQC, and overlapping pair-end sequences were identified and joined using FLASH^33^. Sequences coding rRNA versus ones coding other than rRNA (‘non-rRNA sequences’) were sorted using SortMeRNA^34^ with its built-in reference databases. Non-rRNA sequences in CP and BBD libraries were separately clustered at 97% sequence identity using CD-HIT^20^. Representative sequences of non-rRNA sequences were checked against the NCBI-nr database and UCP database, using the same RapSearch2, BLASTx and MEGAN procedures as the metagenomic analysis detailed previously.

To profile transcriptomic patterns in microbial populations represented by the genomic bins identified, non-rRNA sequences were simultaneously mapped against all bin sequences using BWA (‘bwa mem’ mode)^35^. Average coverages (*i.e.* depths) of transcriptomes mapped on predicted genes in bins were identified using SAMtools (Phred score >25, properly-paired)^36^ and expressed genes within each bin were ranked according to the coverages. Resulting transcriptomic profiles of each metagenomic bin were compared between CP and BBD and shifts in the ranking of expressed genes were identified. To obtain biologically meaningful comparisons of these transcriptomic patterns, genomic bins were required to meet the following conditions: (1) the metagenomic bin had more than 50% gene completeness, as predicted with CheckM^30^, (2) mean coverage for the whole bin was more than 2 in both CP and BBD, and (3) more than 70% of predicted genes in the bin were mapped with transcripts in both CP- and BBD-libraries. The bins Cya1, Cya2 and Oce met these criteria (Table 1).

### Statistical analyses

Proportions of taxonomic and functional terms (calculated as relative abundances (%) within the total number of taxonomically- or functionally-identified sequences)^37^ were compared between CP and BBD datasets, and statistically significant differences were identified with non-parametric bootstrap tests using STAMP (100 times repetition; two-sided comparison; Newcombe-Willson confidence interval method (99.9%); minimum sequence number > 10; Benjamini-Hochberg false discovery rate correction; p-value < 0.001)^38^. Among the terms that showed statistical significance, the most biologically relevant differences were further identified by applying individual filters implemented in STAMP, as appropriate (up to 0.6% of ‘differences between proportions’ and up to 1.5-fold of ‘ratio of proportions’)^39^. Differences of expressed genes in the metagenome-enabled transcriptomic analysis were also statistically tested with a non-parametric bootstrap test using STAMP with the same parameters as above.

### Accession numbers

The raw sequence data used in this study were submitted to the NCBI Sequence Read Archive (SRA) under the accession numbers SRR3499156 and SRR3569370 - SRR3569372 (see Suppl. Table 1).

## Results and Discussion

### Dominance of cyanobacteria and the importance of photosynthesis within black band disease lesions

Over 70-million sequences of DNA and RNA per library were retrieved from CP and BBD lesions, providing comprehensive comparative taxonomic and functional profiles of the lesions (Suppl. Table 2). For taxonomic community profiling, sequences were searched against a collection of universally conserved proteins (UCP; Fig. 2a, Suppl. Data 1 and 2). In both metagenomes and metatranscriptomes, the majority of CP-derived taxonomic marker genes were affiliated to Cyanobacteria, Proteobacteria and Bacteroidetes; BBD-derived taxonomic marker sequences were also dominated by sequences associated with Cyanobacteria and Proteobacteria, but the Bacteroidetes did not represent a major component of BBD communities (Fig. 2a; see Supplementary document for detailed comparisons of sequences taxonomically associated with Bacteria, as well as information on eukaryotic, archaeal and viral-associated sequences). Metagenomic assembly and binning approaches clearly indicated a characteristic shift in the dominant cyanobacterium (*i.e.* Cya1 in CP vs. Cya2 in BBD; Table 1; Suppl. Fig. 1), as has been demonstrated in previous studies^11^,^14^. Complete 16 S rRNA-coding gene sequences in Cya1 and Cya2 shared, respectively, 98% identity with *Trichodesmium erythraeum* IMS101 (GenBank accession NR_074275) and 99% identity with a BBD-dominating cyanobacterium that was previously sequenced from the Caribbean (HM768341) currently identified as *Roseofilum reptotaenium*^40^. Based on the relative sequence abundance of metagenomic bins, the BBD-dominating cyanobacterium Cya2 was approximately 291-fold enriched in the BBD community compared to the CP community (Table 1).

Functional annotation of sequences showed that genes associated with photosynthesis were relatively more abundant in the BBD than in the CP metagenome, and this pattern was even more prominent in the metatranscriptomic comparison (Fig. 2b). Genes associated with CO_2_-fixation were similarly more abundant in both the metagenomes and metatranscriptomes of BBD than in CP (Fig. 2c). The taxonomic affiliations of these CO_2_-fixation sequences in both lesions were dominated by cyanobacteria though further enriched in BBD than in CP (Suppl. Fig. 2a), suggesting that the relative importance of cyanobacteria in CO_2_-fixation increases during the onset of BBD. This is consistent with increased cyanobacterial biomass in BBD compared to CP^15^, and increased cyanobacterial biomass and photosynthetic activity likely result in greater CO_2_-fixation rates within the BBD lesion.

Functional assignments at the lower functional category level (*i.e.* SEED level 3) provided further insights into which microbial drivers are important contributors to BBD virulence, based on higher relative read abundance in the metagenomes and metatranscriptomes of BBD lesions compared to CP lesions. These included photosynthesis clustered genes such as photosystem I (PSI), photosystem II (PSII), light-harvesting complex phycobilisome and chlorophyll-biosynthesis (Fig. 3). Similarly, sequences of genes associated with CO_2_-fixation, specifically involved in the Calvin-Benson cycle and CO_2_-uptake apparatus carboxysome, were more abundant in BBD than in CP. The biosynthesis of fatty acids displayed higher relative read abundance in BBD than in CP (labelled as ‘mycolic acid synthesis’, though taxonomic composition of these sequences indicated that they were predominantly cyanobacterial; data not shown). Within the metagenomic datasets alone, sequences associated with the biosynthesis of some amino acids (glutamine, asparagine and their derivatives) as well as glycogen and ubiquinone biosynthesis were of greater relative abundance in BBD (Fig. 3a) while in the metatranscriptomic datasets F0F1-type ATP synthases demonstrated higher relative abundance in BBD (Fig. 3b). These results demonstrate enhanced anabolic pathways in BBD lesions and suggest that the BBD microbial community has a greater capacity to self-produce organic resources than the CP community, which is likely driven by cyanobacterial photosynthesis and carbon-fixation. In contrast, the higher relative abundance of sequences associated with catabolism-based energy production processes in CP datasets, such as (1) the central energy production pathway, tricarboxylic acid cycle (TCA cycle) and (2) the aerobic respiratory electron transport chain complex, cytochrome C oxidase (Fig. 3), suggests that the microbial communities in CP more actively utilize organic resources aerobically than communities in BBD. Interestingly, sequences related to fermentation were relatively more abundant in BBD than in CP in the metatranscriptomic comparison (Fig. 2c). This greater expression of anaerobic metabolism in BBD lesions is likely linked to the presence of anoxic microenvironmental conditions formed within the BBD lesion^41^, and therefore indicates another factor contributing to the virulence of BBD^15^,^42^ since microbially-mediated anoxia and consequent decrease of pH can rapidly initiate degradation of coral tissues^43^.

### A putative adaptive mechanism of the BBD-dominant cyanobacterium to sulfide accumulation

**Figure 1:**
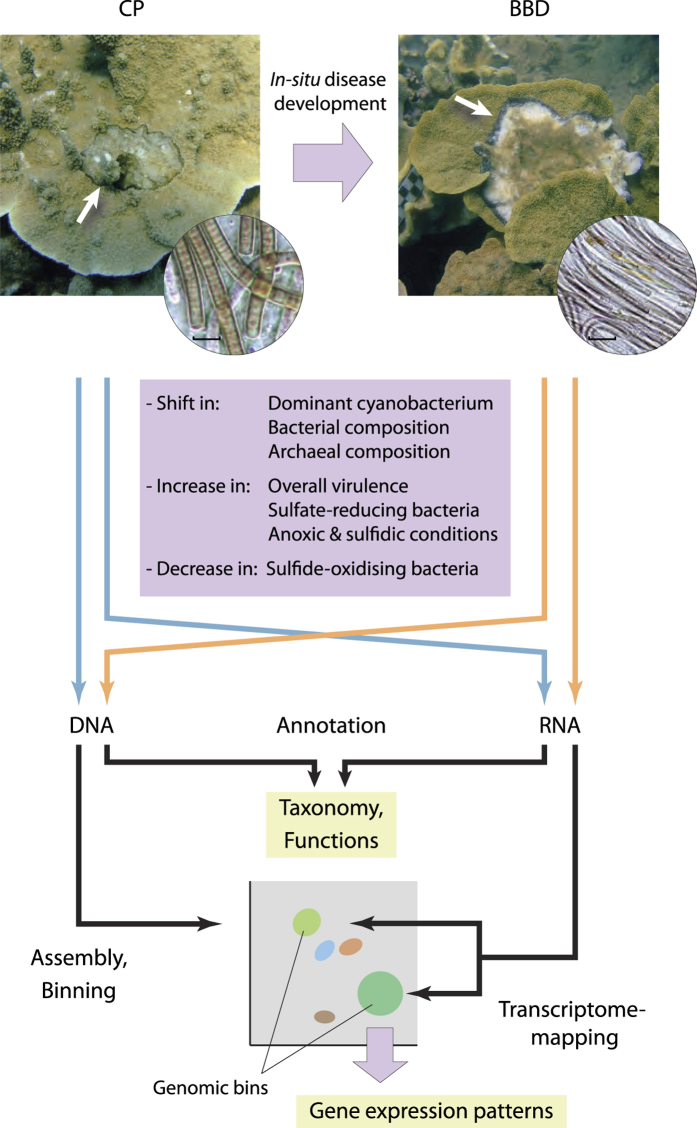
Study concept for elucidating the onset of black band disease (BBD) as it develops from a precursor stage (cyanobacterial patches; CP), using a comparative metagenomics and metatranscriptomics approach. Coral colonies infected with CP were individually monitored in the field as lesions transitioned into BBD. Characteristic changes in microbial communities associated with BBD lesions, as documented in previous studies, are listed on the schematic. DNA and RNA were extracted from microbial mat specimens collected during BBD onset to construct metagenomic and metatranscriptomic libraries, respectively. Resulting metagenomes and metatranscriptomes were annotated taxonomically and functionally. Metagenomic sequences were assembled and binned, and metatranscriptomic sequences were mapped to the metagenomic bins to profile gene expression patterns within the major microbial members. White arrows denote lesions of CP and BBD on coral colonies. Microscopic pictures in circles show the different cyanobacterial-dominated microbial communities associated with lesions of CP and BBD. Scale bars indicate 20 μm.

Metatranscriptomes were mapped to metagenomic bins representing the dominant cyanobacteria in CP (Cya1) and BBD (Cya2) to identify shifts in the gene expression of each cyanobacterial population at each stage of lesion development and gain further insights into the functional roles of Cya1- and Cya2-populations in BBD pathogenesis (Fig. 1). The dominant cyanobacterium associated with BBD, represented by the genomic bin Cya2, demonstrated higher expression of a number of genes involved in carbon concentration (carboxysome) and fixation (ribulose bisphosphate carboxylase, RuBisCo) in the BBD community than in the CP community (Table 2). In contrast, a glycolysis-associated catalase was expressed at lower levels in BBD than in CP (Table 2). This suggests that Cya2 effectively produces carbohydrates by upregulating carbon-fixation within the BBD lesion, while it consumes carbohydrates more actively within the CP-associated microbial mat through glycolysis. A nicotinamide adenine dinucleotide phosphate (NADPH)-quinone oxidoreductase involved in ATP-synthesis was also expressed at greater levels in BBD than in CP, whereas a gene coding phenylalanine-tRNA synthetase (*pheS*), a key checkpoint enzyme for mistranslated proteins due to oxidative damage^44^, was comparatively upregulated in CP lesions, suggesting that Cya2 may actively produce energy in BBD, though experience conditions that are stressful within CP lesions. These observations provide genomic-based evidence that the dominant BBD cyanobacterium is well adapted to microenvironmental conditions within the BBD lesion, which include higher concentrations of sulfide compared to the CP lesion^15^. This is in agreement with the previous metaproteomics study that has indicated that the dominant BBD cyanobacterium expresses proteins for coping with stresses in sulfide-rich environments^45^.

Transcriptomic profiles of the Cya2 bin also show that under microenvironmental conditions of the BBD lesions, Cya2 upregulates a number of genes associated with light-harvesting phycobilisomes, reaction-center subunits of PSI, and electron-transfer in photosynthesis, but downregulates genes coding PSII reaction center proteins and PSI apoproteins (but not reaction center proteins; Table 2). These results indicate the presence of a putative adaptive mechanism, by which the BBD-cyanobacterium copes with the accumulation of sulfide within BBD lesions. Previous culture-based physiological tests on the BBD-dominating cyanobacterium, *R. reptotaenium*^40^, have demonstrated that it is capable of sulfide-tolerant oxygenic photosynthesis^46^, which is similar to a capability of cyanobacteria occurring in sulfidic spring biofilms that have daily fluctuations in light and sulfide^47^. In general, sulfide can negatively affect functioning of the oxygen-evolving complex at the initial water-splitting step in PSII^48^, but a certain amount of sulfide (*e.g.* up to 210 μM) can also improve the gross photosynthesis of some cyanobacteria^47^. A kinetic regulation model based on the study of *Planktothrix* sp. suggests that the cyanobacterium overcomes the inhibitory effect of sulfide by enhancing light-harvesting capabilities of PSII and PSI, increasing electron transport efficiencies downstream of PSII (*e.g.* plastocyanin), and increasing the number of PSI reaction centers in the thylakoid membrane^47^. Sulfide also stimulates upregulation of genes involved in the PSI and the carbon-fixing Calvin cycle, such as RuBisCo, thereby affecting photosystem stoichiometry (PSII vs. PSI)^49^,^50^. The gene-expression patterns of Cya2 bear a striking similarity to responses of other sulfide-adapted photosynthetic organisms (Fig. 4), strongly suggesting that the BBD-dominating cyanobacterium possesses the capacity for metabolic adaptation to sulfidic environments by adopting a similar strategy. The expression of the *sqr*-gene that governs sulfide-driven anoxygenic photosynthesis was also detected in the Cya2 bin providing further evidence for this adaptive mechanism (see Supplementary document for additional discussion on the Cya2’s capabilities). Importantly, the CP-dominating cyanobacterial bin (Cya1) did not display this pattern in transcriptomic gene expression associated with photosynthetic apparatus (Table 2), indicating that the CP-dominating cyanobacterium is not adapted to the sulfidic conditions that characterize BBD lesions, and thus likely outcompeted by the BBD-dominating cyanobacterium during development of BBD pathogenicity.

### Cyanobacterial photosynthates support sulfide production by Deltaproteobacteria

**Figure 2:**
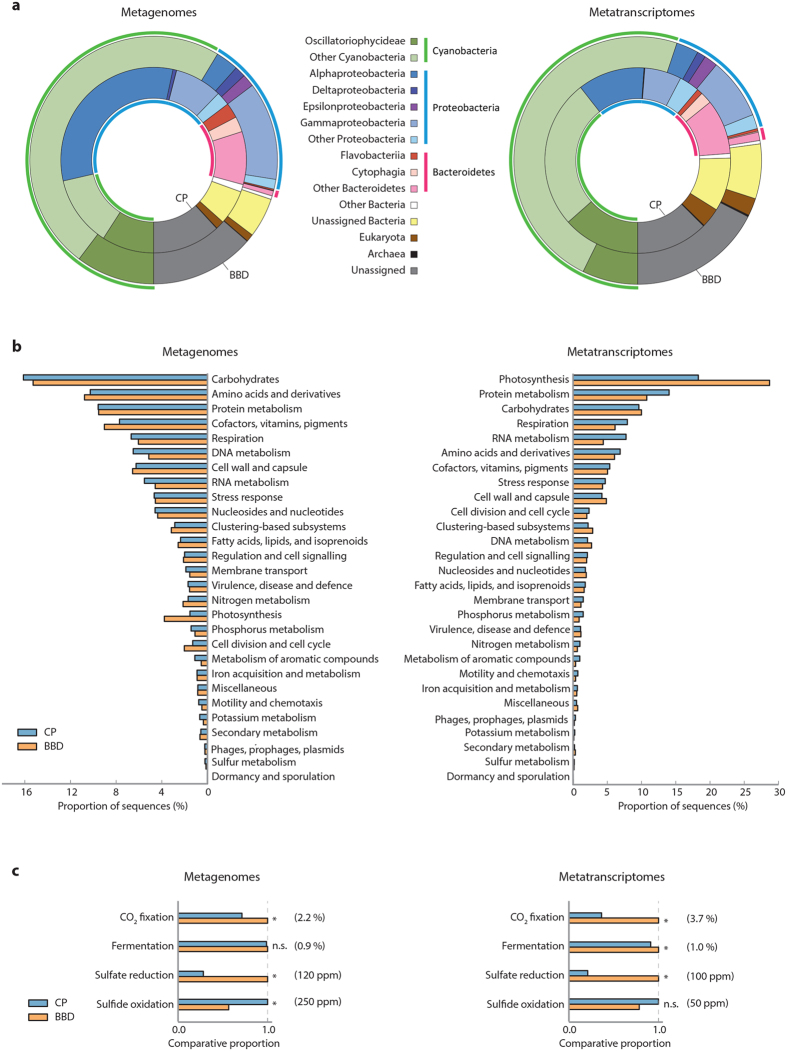
Comparisons of overall profiles of metagenomes and metatranscriptomes recovered from microbial lesions of cyanobacterial patches (CP) and black band disease (BBD). (**a**) Composition of taxonomically annotated sequences in the CP (inner graphs) and BBD (outer graphs) datasets based on the BLAST search against the universally conserved proteins database. (**b**) Functional profiles classified at a high hierarchy level (SEED Subsystem level 1). (**c**) Relative abundance of sequences affiliated with genes involved in selected functions highlighted in this study: CO_2_-fixation (SEED level 2 term “CO_2_ fixation”), fermentation (SEED level 2 term “fermentation”), sulfate reduction (SEED level 2 term “sulfate-reduction associated complexes”) and sulfide oxidation (SEED level 2 term “sulfur oxidation”). Horizontal bars compare relative proportions of sequences between CP and BBD, with whichever is higher being set as 1.0. Actual proportions of the higher value (in either CP or BBD) are indicated in brackets, calculated as the relative sequence abundance within all sequences that were assigned to SEED Subsystems terms. Asterisks, p < 0.001; n.s., non-significant; ppm, parts per million.

**Figure 3:**
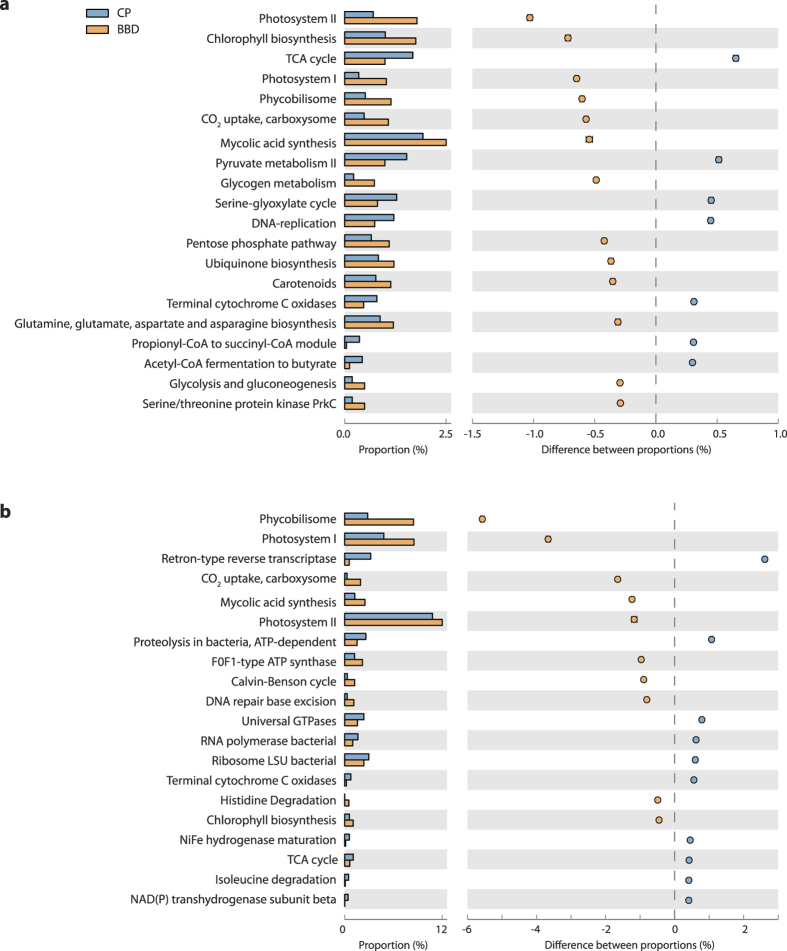
The 20 most significantly different items (p < 1e-100) in functional comparisons of metagenomes (**a**) and metatranscriptomes (**b**) recovered from microbial lesions of cyanobacterial patches (CP) and black band disease (BBD) at a lower hierarchy level (SEED Subsystem level 3). Left histogram: relative proportions; Right figure: differences between proportions (negative values indicate BBD > CP). Error bars denote 99.9% confidence intervals (most not visible due to small error ranges).

**Figure 4:**
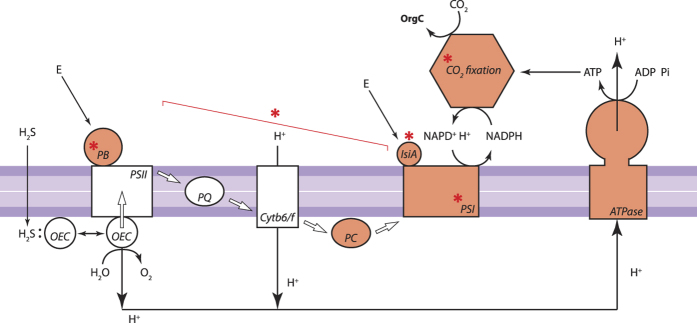
Schematic illustration showing gene-expression pattern in photosynthetic-related apparatus of the Cya2 genomic bin dominating black band disease (BBD). Orange-colored components indicate prominently higher expression of genes within BBD lesions than in cyanobacterial patches (CP). Red asterisks denote components observed or predicted to be enhanced during photosynthetic adaptation to sulfide accumulation by previous studies^47^,^49^,^50^. White arrows indicate electron flow between components. PSI: photosystem I; PSII: photosystem II; OEC: oxygen evolution complex; PB: phycobilisome; E: photon flux; PQ: plastoquinone; Cytb6/f, cytochrome b6/f complex; PC: plastocyanin; IsiA: iron-limitation induced chlorophyll-binding protein; ATPase: ATP synthase; OrgC: organic carbon compounds. Illustration is adapted from the kinetic control model in Klatt *et al*.^47^.

The accumulation of sulfide within lesions has been implicated in the development of BBD pathogenicity^15^,^42^,^51^. Previous quantitative PCR studies targeting representative functional genes in sulfate-reduction (*dsrA*) and sulfide-oxidation (*soxB*) pathways have demonstrated that sulfate-reducers are more abundant, whereas sulfide-oxidizers are less abundant in BBD than in CP lesions^12^,^13^. In the present study, a comprehensive list of sulfur-cycling genes was investigated in microbial consortia and confirmed higher sulfate-reduction and lower sulfide-oxidation in BBD than CP, both in the metagenomes and metatranscriptomes (Fig. 2c). Sequences associated with sulfate-reduction in BBD were taxonomically dominated by Deltaproteobacteria, whereas Gammaproteobacteria dominated in CP (Suppl. Fig. 2b). The majority of these deltaproteobacterial sequences in BBD were affiliated to *Desulfovibrio* spp., previously proposed as the main sulfide-producer in BBD lesions^51^. The higher abundance of sulfate-reducing sequences in BBD is also consistent with the higher relative abundance of Deltaproteobacteria in the overall taxonomic composition of BBD communities than in CP communities (Fig. 2a). Furthermore, within deltaproteobacterial sequences coding for degradation and utilization of organic compounds, those for carbohydrates were more highly expressed compared to those for amino acids, fatty acids and proteins (Suppl. Fig. 3). This strongly suggests that Deltaproteobacteria in BBD lesions are relying on carbohydrates as energy substrates. Since the BBD cyanobacterium is the predominant carbon fixer in BBD lesions (Suppl. Fig. 2a), we hypothesize that Deltaproteobacteria utilize carbohydrates of cyanobacterial origin as part of their required electron donors to gain energy by sulfate-reduction and thus there is a link between cyanobacterial photosynthesis and sulfide-production by Deltaproteobacteria.

### Other aspects of microbial functioning within lesion communities

Previous studies have proposed that accumulated sulfide in BBD lesions is utilized by sulfide-oxidizers, which have been identified as *Beggiatoa* spp. (Gammaproteobacteria) and members of the Rhodobacteraceae family (Alphaproteobacteria), based on a light microscopy study^52^ and taxonomic profiling of a representative sulfide oxidation gene (soxB)^13^, respectively. However, taxonomic annotation of whole sulfide-oxidization genes in our metagenomic and metatranscriptomic datasets indicated that sulfide-oxidization genes are predominantly expressed by Epsilonproteobacteria in the BBD lesion, but primarily by Alphaproteobacteria in CP (Suppl. Fig. 2c). Although epsilonproteobacterial sequences were more abundant in BBD than CP within the lesion communities (Fig. 2a, Suppl. Fig. 4), it is important to note that the total abundance and expression of sulfide-oxidizing genes were higher in CP (Fig. 2c). Our results therefore provide further confirmation that sulfide-oxidation is not an integral part of the development of BBD virulence; instead, reduced capacity of sulfide-oxidation further contributes to the accumulation of hydrogen sulfide within the BBD lesion^13^. *Arcobacter* spp., which are obligate sulfide-oxidizing autotrophs commonly occurring in marine redoxclines where hydrogen sulfide and oxygen are available^53^,^54^, were the dominating species in the BBD-associated Epsilonproteobacteria community (Suppl. Data 1 and 2). Taken together the accumulated hydrogen sulfide within BBD lesions creates ecological niches for specific sulfide oxidizers such as *Arcobacter* species, but these organisms are likely secondary colonizers since sulfide-oxidization does not appear to be directly linked to BBD pathogenicity.

The composition of sequences associated with degradation and utilization of various organic matter types differed among the major non-photosynthesizing bacterial taxa (Suppl. Fig. 3), suggesting that compositional changes in the available organic substrates within CP and BBD lesions are in part responsible for shifts in microbial community profiles. Transcriptomes mapped against the Oce-bin, affiliated to the gammaproteobacterial order Oceanospirillales (Table 1), displayed gene expression trends indicating that this population aerobically degrades lipids more actively within the BBD lesion than in the CP lesion. Their relative increase in lipid-metabolism within the BBD lesion is potentially resulting from increased total lipids derived from degrading cyanobacterial cell walls and/or coral tissue. Transcriptomic patterns in Oce also suggest a potential link between the enhanced aerobic lipid metabolism and enhanced expression of ATP-production and virulence factors within BBD lesions. These oxygen-consuming metabolisms pathways likely promote formation of an anoxic microenvironment within the BBD lesion especially during the dark period without cyanobacterial oxygenic photosynthesis, further contributing to the virulence of BBD (Suppl. Table 3; see Supplementary document for details). The involvement of bacteria with known pathogenic traits (*e.g.* toxin-producing cyanobacteria, *Vibrio, Cytophaga, Clostridium* and *Campylobacter* species) in BBD virulence has also been suggested based on 16 S rRNA gene sequence similarities^55^,^56^,^57^. In particular, cyanotoxins and *Vibrio*-related toxins have been proposed to play roles in BBD pathogenicity^56^,^58^. Our data, however, showed that sequences associated with these virulence factors were not consistently more abundant and/or expressed in the metagenome and metatranscriptomes of BBD compared to those of CP (see Supplementary document for details). The present study thus suggests that cyanotoxins and *Vibrio*-virulence are not major microbial drivers in the development of BBD virulence.

## Conclusions

**Figure 5:**
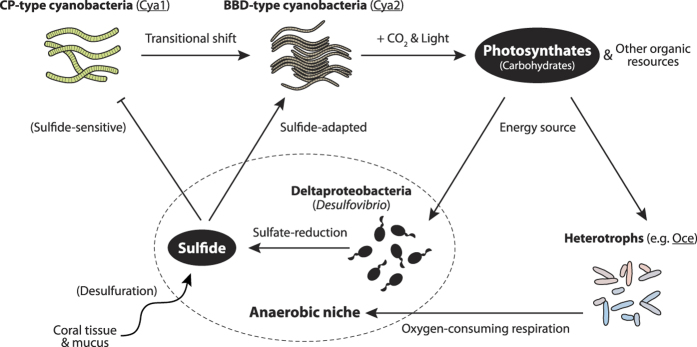
Concept model of black band disease (BBD) onset showing key microbial drivers according to sequence-based evidence. Increased photosynthesis by the BBD-type cyanobacterium (Cya2) drives organic material synthesis and the respiration metabolism of heterotrophs, such as gammaproteobacterial populations (*e.g.* Oce). Anaerobic microbial environment is created by oxygen-consuming respiration of heterotrophs. Within the anaerobic niche, cyanobacterial photosynthates drive sulfate-reduction by deltaproteobacterial sulfate-reducers, producing sulfide within the polymicrobial lesion, with possible further contributions from desulfuration of degrading coral tissue and mucus. Cyanobacteria associated with cyanobacterial patches (CP; Cya1) become outcompeted by Cya2, which is able to adopt a sulfide-adaptation mechanism, facilitating the transition between CP and BBD. Resulting anoxic, sulfide-rich microenvironmental conditions contribute to increased pathogenicity to underlying coral tissue, which especially intensifies during dark-periods compared to light conditions.

Recent advances in high-throughput sequencing technologies and bioinformatic methodologies have enabled an improved understanding of coral diseases by profiling genomes and transcriptomes of host, symbionts and potential pathogens^59^,^60^,^61^. The integrated genomic approach applied in this study documented the differences in microbial function between comparatively benign CP lesions and virulent BBD lesions, providing in-depth insights into the microbial mechanisms responsible for the development and progression of BBD in corals. These results highlight the pivotal role of the BBD-dominating cyanobacterium in the development of overall virulence of the polymicrobial disease lesion (Fig. 5), specifically its role in increasing CO_2_-fixation and biosynthesis of organic compounds during disease onset, driven by high levels of photosynthetic activities. The BBD microbial community thus appears to have a metabolic repertoire that generates its own organic resources within the lesion, explaining how a BBD infection can persist for up to several months in corals under natural conditions^16^. Increased organic resource production likely supports further growth of heterotrophic bacterial populations in BBD, which in turn fuels their oxygen-consuming catabolism. These metabolic activities can create the anaerobic microenvironments found at the bottom of microbial mats, where light and oxygen do not penetrate^15^,^41^, providing the niche required for obligate or facultative anaerobes, as evidenced by relative increases in sequences affiliated to anaerobic sulfate-reducers and fermentative metabolisms. Sulfate-reducing Deltaproteobacteria, dominated by *Desulfovibrio* species, appear to predominantly degrade and utilize carbohydrates among other organic materials, suggesting that increased cyanobacterial photosynthates are at least in part responsible for the increase of sulfide within the polymicrobial lesion.

The current study did not identify the flux rate of microbial sulfide-production, and it is important to highlight that sulfide released by abiotic and biotic desulfuration of degraded coral tissue and mucus may also contribute to the accumulation of sulfide within BBD lesions^43^,^62^. Further investigations are thus required to identify the source(s) of sulfide production using more direct methods, such as isotope element tracing and microbial sulfur-influx measurement. Weber *et al*.^43^ detailed that sediments on coral decrease oxygen and pH at the tissue interface. Reduced oxygen and pH in combination can rapidly initiate coral tissue necrosis, which is further accelerated by accumulating sulfide. The presence of anoxic and sulfidic microenvironment within the BBD mat suggests that a similar mechanism may be involved in BBD virulence. Decreases in oxygen and pH as well as an increase of sulfide within the BBD lesion become highly prominent during the dark compared to the light period when cyanobacteria perform oxygenic photosynthesis^15^. Although the current study collected specimens during the day, results do provide evidence for increased expression of metabolic pathways characteristic of anaerobic, sulfide-rich environments. Metatranscriptomic comparison of BBD-associated microbial communities between day and night would however facilitate a greater understanding of the microbial mechanisms underlying the pathogenicity of BBD.

This study also provides sequence-based evidence of a putative adaptive mechanism evolved by the dominant BBD cyanobacterium to handle sulfide accumulation within lesions, improving the current mechanistic understanding of BBD pathogenesis. Our results highlight the central role that the dominant cyanobacterium has in BBD pathogenesis, providing the structural foundation for a stratified, dynamic microenvironment within lesions, with sulfide-tolerance being a key attribute enabling it to establish an ecological niche in the sulfide-rich mat, similar to other cyanobacteria found in sulfidic environments^48^. Given the ancient nature of sulfide-rich environments^63^, further study of molecular mechanisms underpinning the capacity of this cyanobacterium to flourish in BBD lesions may reveal its evolutionary roots in terms of how it has interacted with reef environments and potentially illuminate the origin of this widespread coral disease. The microbial communities associated with BBD display strikingly similar community structures among global locations^64^. This consistent nature of BBD highlights that our findings based on this CP-BBD model system contribute to a wider understanding of the microbial mechanisms responsible for BBD pathogenicity because different biological and physical triggers, such as organic-rich sedimentation^43^, can create microbial niches that are required for the development of BBD. The present study therefore presents new sequence-based evidence derived from whole *in-situ* disease lesions that underpins microbial metabolism and interactions during the onset and progression of BBD, further supporting a previously proposed concept model of BBD pathogenesis^10^.

## Additional Information

**How to cite this article**: Sato, Y. *et al*. Unraveling the microbial processes of black band disease in corals through integrated genomics. *Sci. Rep.* **7**, 40455; doi: 10.1038/srep40455 (2017).

**Publisher's note:** Springer Nature remains neutral with regard to jurisdictional claims in published maps and institutional affiliations.

## Supplementary Material

Supplementary Document

Supplementary Data 1

Supplementary Data 2

Supplementary Data 3

Supplementary Data 4

Supplementary Data 5

## Figures and Tables

**Table 1:** Summary statistics of metagenomic binning and metagenome-enabled transcriptomics based on sequence data recovered from microbial lesions associated with cyanobacterial patches (CP) and black band disease (BBD).

Bin ID	Taxonomic assignment*^1^	Bin size (bp)	Metagenomes						Metatranscriptomes			
			GC (%)*^2^	Complete-ness (%)*^3^	Contami-nation (%)*^3^	Predicted genes	Relative abund. In CP (%)*^4^	Relative abund. In BBD (%)*^4^	Mean coverage in CP	Mean coverage in BBD	Mapped genes in CP*^5^	Mapped genes in BBD*^5^
Alpha1	Alphaproteobacteria	455,413	63.5	12.66	0.4	596	0.50	0.06	10.89	1.13	581 (97%)	424 (71%)
Alpha2	Alphaproteobacteria	509,736	61.4	21.42	2.1	616	0.40	0.02	7.95	2.10	553 (90%)	353 (57%)
Alpha3	Alphaproteobacteria	4,014,180	46.5	94.78	1.7	3981	1.70	0.02	3.87	0.06	3522 (88%)	591 (15%)
Alpha4	Rhodobacteraceae	15,466	59.0	3.21	1.3	17	0.02	0.00	8.77	9.30	15 (88%)	16 (94%)
Alpha5	Rhodobacteraceae	91,334	60.5	10.82	1.7	100	0.10	0.00	4.62	0.91	92 (92%)	71 (71%)
Alpha6	*Ruegeria*	122,708	58.5	4.17	0.0	138	0.09	0.00	0.65	0.77	92 (67%)	91 (66%)
Alt1	Alteromonadales	2,417,030	41.8	51.51	8.4	2718	0.43	0.55	1.26	3.05	2004 (74%)	2312 (85%)
Alt2	Alteromonadaceae	3,953,240	41.4	81.06	3.9	3735	1.39	0.75	0.83	1.80	2727 (73%)	3093 (83%)
Alt3	Alteromonadales	76,910	44.8	3.45	0.9	74	0.01	0.01	0.68	1.52	30 (41%)	33 (45%)
Alt4	Gammaproteobacteria	24,749	47.4	1.65	0.1	38	0.00	0.01	1.34	31.74	33 (87%)	35 (92%)
Cam	*Arcobacter*	573,805	27.8	27.33	0.4	659	0.00	0.10	0.23	3.70	273 (41%)	572 (87%)
Cya1	*Trichodesmium erythraeum*	5,081,837	35.4	60.2	4.3	6597	16.21	0.13	41.62	2.14	6244 (95%)	4630 (70%)
Cya2	Cyanobacteria*^6^	5,612,706	44.8	99.33	0.2	5452	0.19	55.32	2.61	203.06	4622 (85%)	5327 (98%)
Cyt1	Bacteroidetes	6,703,172	33.5	96.22	3.8	6866	5.98	0.04	3.58	0.32	5930 (86%)	3456 (50%)
Cyt2	Cytophagales	3,642,844	40.1	86.58	7.4	3872	0.92	0.00	2.88	0.03	3280 (85%)	384 (10%)
Cyt3	Cytophagales	7,396,682	35.3	94.91	2.7	6385	3.08	0.00	1.28	0.02	4950 (78%)	771 (12%)
Fla1	Flavobacteriaceae	1,944,701	34.9	60.98	4.4	2172	0.48	0.01	0.16	0.31	869 (40%)	1013 (47%)
Fla2	Flavobacteriaceae	995,165	33.0	35.2	0.9	1112	0.21	0.03	0.58	0.13	673 (61%)	385 (35%)
Oce	Gammaproteobacteria	4,647,524	46.0	98.71	18.3	4479	0.55	1.29	6.11	3.65	3923 (88%)	4031 (90%)

^*1^Based on assignment of the lowest common taxonomic level of taxonomic markers detected with AMPHORA.

^*2^Guanine-cytosine content.

^*3^Based on taxonomic marker sets determined with CheckM.

^*4^Relative abundance of the genomic bin, calculated as a proportion of metagenomic reads mapped to a bin in the total number of reads in the metagenomic library.

^*5^The number is indicated as the number of predicted genes that were mapped with transcriptomes. The proportion was calculated by dividing the number by the total number of predicted genes in the bin.

^*6^A complete 16 S rRNA coding gene in Cya2 was assigned as the BBD-dominating cyanobacteria that were previously sequenced (99% nucleotide identity over 1,470-bp; HM768341), classified as the species *Roseofilum reptotaenium*.

**Table 2:** Metagenome-enabled transcriptomic analysis on the genomic bins associated with the dominant cyanobacteria in black band disease (BBD; Cya2) and cyanobacterial patches (CP; Cya1).

Bin	BBD>CP			CP>BBD		
	General functions	Protein-coding gene assignments	Rank*	General functions	Protein-coding gene assignments	Rank*
Cya2	Light harvesting	Phycobilisome C-phycocyanin alpha chain (cpcA)	+7	Photosystem II	Photosystem Q(B) reaction center protein (psbA; D1 protein)	−2
		Phycobilisome C-phycocyanin beta chain (cpcB)	+27		Photosystem Q(B) reaction center protein 1 (psbA; D1 protein)	−4
		Phycobilisome C-phycoerythrin alpha chain (cpeA)	+2		Photosystem II CP43 reaction center protein (psbC)	−54
		Phycobilisome C-phycoerythrin class 1 subunit beta (cpeB)	+7		Photosystem II reaction center protein H (psbH)	−630
		Phycobilisome 27.9 kDa linker polypeptide (cpeD)	+32	Photosystem I	Photosystem I P700 chlorophyll a apoprotein A1 (psaA)	−17
		Phycobilisome rod-core linker polypeptide (cpcG)	+107		Photosystem I P700 chlorophyll a apoprotein A2 (psaB)	−16
	Photosystem I	Photosystem I iron-sulfur center (psaC)	+8	Light harvesting	Allophycocyanin beta chain (apcB)	−5
		Photosystem I reaction center subunit III (psaF)	+11	Glycolysis	Fructose-bisphosphate aldolase class 2 (fbaA)	−171
		Photosystem I reaction center subunit IV (psaE)	+8	Protein synthesis/quality control	Phenylalanine-tRNA synthetase (pheS)	−790
		Photosystem I reaction center subunit IX (psaJ)	+22			
	Carbon fixation	Ribulose bisphosphate carboxylase large chain (RuBisCO; rbcL)	+46			
		Ribulose bisphosphate carboxylase small chain (RuBisCo; rbcS)	+235			
	Carbon concentrating	Carbon dioxide-concentrating mechanism protein (carboxysome, ccmK)	+167			
	Photosynthesis e^-^-transfer	Plastocyanin (petE)	+197			
	ATP synthesis	NAD(P)H-quinone oxidoreductase chain 4 1 (ndhD1)	+667			
	Heavy metal resistance	Metallothionein (smtA)	+51			
	Iron limitation resistance	Iron-limitation induced chlorophyll-binding protein (isiA)	+134			
Cya1	Light harvesting	C-phycocyanin beta chain (cpcB)	+15	Photosystem I	Photosystem I iron-sulfur center (psaC)	−21
		R-phycocyanin−2 subunit alpha (cpcA)	+31		Photosystem I reaction center subunit (psaK)	−102
	Photosystem II	Photosystem II 32 kDa reaction center protein (psbA; D1 protein)	+2		Photosystem I reaction center subunit III (psaF)	−44
	Vitamin B6 synthesis	Pyridoxine/pyridoxamine 5’-phosphate oxidase 2 (ppox2)	+155		Photosystem I reaction center subunit IV (psaE)	−13
	Translation	50 S ribosomal protein L3 (rplC)	+325	Carbohydrate degradation	6-phosphogluconolactonase (pgl)	−1429
				Protein folding	10 kDa chaperonin (groS)	−381

General functions and protein-coding gene assignments that were differentially expressed in the genomic bins are shown with differences in rankings between CP and BBD. Tables show predicted genes that are the most expressed (top 100 in either CP and/or BBD libraries) and their expressions are significantly different between CP and BBD (p < 0.001; bootstrap tests). Only genes assigned proteins with well-characterized specific functions are shown, and hypothetical genes and genes associated with unspecific function(s) were excluded.

*Rank difference: Positive values indicate that the organism represented by the bin had relatively higher proportions of transcriptomes mapped to the corresponding gene in the BBD library compared to the CP library, and negative values (−) indicate vice versa.
