# Supplementary material for: Unraveling the microbial processes of black band disease in corals through integrated genomics
Source: Sci Rep. 2017 Jan 17;7:40455. doi: 10.1038/srep40455 (PMC5240343; doi:10.1038/srep40455)
Supplement: Supplementary Document [file srep40455-s1.pdf]

Supplementary document for;

Unraveling the microbial processes of black band disease in corals through integrated genomics

Yui Sato[1], Edmund Y. S. Ling[2], Dmitrij Turaev[3], Patrick Laffy[1], Karen D. Weynberg[1], Thomas Rattei[3], Bette L. Willis[4,5], David G. Bourne[1,5 *]

[1] Australian Institute of Marine Science, PMB 3, Townsville MC, Townsville 4810, Australia

[2] Global Change Institute, School of Agriculture & Food Sciences, The University of Queensland, Brisbane 4072, Australia

[3] Department of Computational Systems Biology, University of Vienna, Althanstrasse 14, 1090 Vienna, Austria

[4] ARC Centre of Excellence for Coral Reef Studies, James Cook University, Townsville 4811, Australia

[5] Marine Biology and Aquaculture, College of Science and Engineering, James Cook University, Townsville 4811, Australia

This document contains;

1. Supplementary results and discussion
2. Supplementary methods
3. Supplementary figures and tables.

1. Supplementary results and discussion

Bacterial community structures of microbial lesions associated with cyanobacterial patches and black band disease

In both metagenomic and metatranscriptomic datasets derived from polymicrobial lesions of cyanobacterial patches (CP) and black band disease (BBD), sequences assigned to Cyanobacteria, Gammaproteobacteria, Epsilonproteobacteria and Deltaproteobacteria were more abundant in BBD than in CP, while those assigned as Alphaproteobacteria, Cytophaga, Flavobacteriia and other Bacteroidetes were less abundant in BBD than in CP (Figure 2a, Suppl. Figure 4, Suppl. Data 1 and 2). These patterns in bacterial sequence compositions were highly consistent with those in previous PCR-based microbial profiling studies (Sato *et al.* 2010, Sato *et al.* 2013). Specifically, a shift in the dominant cyanobacterial types and increase in relative abundance of sequences associated with Epsilonproteobacteria (dominated by *Arcobacter* spp.) and Deltaproteobacteria (dominated by *Desulfovibrio* spp.) are common features during transition from CP to the BBD stage of pathogenesis. Overall consistency in the taxonomic compositions between metagenomic and metatranscriptomic libraries suggests that taxonomic compositions present as genomic materials (*i.e.* DNA) in the polymicrobial lesion specimens closely represent the compositions of taxa that are actively transcribing functional genes in the community and *vice versa*. Interestingly, within the CP libraries, cyanobacterial sequences were under-represented and alphaproteobacterial sequences over-represented in the metagenomic data compared to the metatranscriptomic data. Although these patterns suggest that the presence of DNA may not always accurately reflect relative functional importance for certain taxonomic groups in a complex microbial community, a detailed reason for why only these taxa displayed such patterns remains unclear.

Cyanobacterial sequences dominating the microbial communities of CP and BBD showed a clear shift from a *Trichodesmium*-affiliated cyanobacterium in CP to a cyanobacterium affiliated to *Roseofilum reptotaenium* in BBD, consistent with previous studies (Sato *et al.* 2010, Sato *et al.* 2013). Importantly, complete 16S rRNA gene sequences recovered from the assembled genomic bins of these cyanobacteria (*i.e.* Cya1 in CP and Cya2 in BBD) confirmed their phylogenetic positions closely matching with cyanobacterial sequences previously retrieved from CP- and BBD-associated cyanobacteria on the GBR (Sato *et al.* 2010; Suppl. Figure 5). The sequence of the BBD-cyanobacteria obtained in this study was also positioned within a tightly clustered group including *R. reptotaenium* and other cyanobacterial sequences retrieved from BBD lesions collected from a wide geographic range including the Caribbean, Rea Sea and Indo-Pacific regions (Supp. Figure 5). There are other divergent cyanobacterial sequences that have been recovered from BBD lesions although some of them constitute only minor components within BBD-associated microbial communities (Myers *et al.* 2007, Sato *et al.* 2013). Interestingly, a few of these sequences (*e.g.* AY123040, DQ446127) form a monophyletic cluster with the CP-dominating cyanobacterial sequences (Suppl. Figure 5). This suggests that a similar successional development of BBD derived from CP-like lesions also occurs in other geographical locations although CP lesions have thus far been reported only from the GBR.

Gammaproteobacterial sequences were also present as an abundant group in both CP and BBD though relatively more abundant in BBD. The compositions of gammaproteobacterial sequences were dominated by those that were not clearly assigned to any identified order, but also including Alteromonadales- and Oceanospirillales-associated sequences in both CP and BBD lesions (Suppl. Data 1 and 2), displaying a pattern also observed in previous sequence-based studies (Sato *et al.* 2010, Sato *et al.* 2013). Gammaproteobacterial-assigned transcriptomes indicated that Gammaproteobacteria in BBD

lesions utilize diverse metabolic substrates including proteins, carbohydrates, amino acids and fatty acids (Suppl. Figure 3). This versatile resource utilization capability within this group may explain the consistent presence of Gammaproteobacteria during the transition from CP to BBD without dramatic changes in the taxonomic compositions while available organic materials and microenvironmental conditions change.

Other taxonomic groups including Archaea, Eukaryota, and viruses within the lesions

Eukaryotic sequences accounted for between 1 and 3% of the total taxonomically-assigned sequences in CP and BBD metagenomes and metatranscriptomes, based on the search against the Universally Conserved Proteins (UCP) database (Figure 2a, Suppl. Data 1 and 2). CP and BBD sequences in metagenomes and metatranscriptomes were assigned to diverse eukaryotic taxa, including Bacillariophyta, Chordata, Streptophyta and Ciliophora (Suppl. Figure 6). Large proportions of these sequences, however, especially in metagenomes, could not be confidently assigned to a defined phylum or had weak support (bit scores < 51) in searches against the UCP database. Archaeal-affiliated sequences represented between 0.1 and 0.2 % of the total taxonomically-assigned sequences across the datasets. Sequences assigned to an unassigned archaeal class, unclassified Thaumarchaeota (including Nitrosopumilales), and the classes Methanopyri and Thermoprotei were more abundant in CP than BBD metagenomes, though archaeal metatranscriptomic sequences displayed inconsistent relative abundance patterns, with an unassigned archaeal class being relatively more abundant in BBD than CP metatranscriptomes (Suppl. Figure 7). In addition, these sequence abundance patterns were not consistent with previous 16S rRNA gene profiling of BBD and CP archaeal communities, which detected a dominant uncultured archaeon belonging to an unique lineage remotely associated with Euryarchaeota (Sato *et al.* 2013).

Overall, the results of eukaryotic and archaeal sequence compositions indicate a potential shortcoming of meta-'omics' approaches for profiling rare members of these microbial communities, due to a lack of sequence information in the publically available sequence databases that represents numerous understudied and unidentified environmental microorganisms (Rinke *et al.* 2013).

CP and BBD datasets contained between 0.02 and 0.04% of sequences taxonomically assigned to viruses (Suppl. Data 4 and 5). Viral-associated metagenomic sequences were mostly dsDNA viruses in both CP and BBD, while viral metatranscriptomic sequences included dsDNA, ssDNA, and positive-sense ssRNA viruses in CP, as well as unclassified environmental viruses that accounted for approximately 67% of BBD-viral sequences (Suppl. Figure 8). These observations reflect the difference in the nucleic acid types that were extracted and sequenced (*i.e.* DNA vs. RNA), but also highlight that since viruses can consist of dsDNA, ssDNA, dsRNA and ssRNA genomes, substantial biases can be introduced when investigating environmental samples by adopting different ribonucleotide targeted approaches (Weynberg *et al.* 2014, Wood-Charlson *et al.* 2015). Sequences assigned to the family *Caudovirales* accounted for the largest proportions in the viral-associated sequences of the CP-derived metagenome and metatranscriptome as well as BBD-derived viral metagenomic sequences, while the viral sequences in the BBD metatranscriptome consisted of a high relative abundance of uncultured cyanophage-associated sequences. Interestingly, these cyanophage-assigned sequences shared ≥99% sequence identity (over 104 nt) with 2 genomic regions coding the PSII D1-protein within the Cya2 genomic bin that represented the dominant cyanobacteria in BBD. We thus hypothesize that the dominant cyanobacterium in BBD has a host-phage association with a novel type of cyanophage, which encodes the PSII gene likely acquired from the host (Lindell *et al.* 2004, Sullivan *et al.* 2006, Chénard and Suttle 2008). Furthermore, the BBD metagenome had relatively more viral sequences than

CP that were associated with *Cellulophaga* phage phiST, which infects members of the *Bacteroidetes* (Holmfeldt *et al.* 2013; Suppl. Figure 8), a bacterial group that was relatively abundant in CP but not well-represented in BBD (Figure 2a). This may indicate that these phages removed *Bacteroidetes* in CP lesions and remained highly abundant in the BBD lesions. Characterization of viral communities associated with coral diseases is becoming increasingly important. Viruses can be considered not only as disease-causing agents affecting a coral host and its symbionts (*e.g.* symbiotic algae *Symbiodinium* spp.) (Pollock *et al.* 2014, Soffer *et al.* 2014, Correa *et al.* 2016), or phenotypes in bacterial pathogens thereby indirectly triggering coral disease (Weynberg *et al.* 2015), but also as therapeutic agents to control coral diseases by lysing pathogenic bacteria (Atad *et al.* 2012, Cohen *et al.* 2013). The presence of the dominant cyanophage-associated sequences in the BBD metatranscriptome highlights a potential option for phage-therapy. However, further investigations are required to characterize the role of viruses in the disease dynamics before we can consider such therapy strategies as a potential tool for mitigating impacts of BBD.

A potential adaptive mechanism within the BBD-dominating cyanobacterium

Data presented here indicate that the BBD-dominating cyanobacterium may perform anoxygenic photosynthesis as an additional mechanism to adapt to increased sulfide concentrations in the BBD lesion. Gene expression patterns of the genomic bin Cya2, representing the BBD-dominating cyanobacteria, suggested the presence of a putative sulfide-adaptive mechanism in their photosynthetic regulation, where genes coding photosystem I (PSI) were highly expressed over those coding photosystem II (PSII; Figure 4a). An alternative explanation for this observed pattern is that the BBD-dominating cyanobacterium possibly performs PSI-driven anoxygenic photosynthesis using sulfide as an

electron donor (Cohen *et al.* 1986, Miller and Bebout 2004), and thus PSII appears to be downregulated in comparison to the expression of PSI reaction center. Interestingly, sequences of a sulfide-quinone reductase (*sqr*), which drives anoxygenic photosynthesis in cyanobacteria (Arieli *et al.* 1994), is present in the Cya2 genomic bin and transcriptomes mapped to the *sqr* sequence were approximately 17-fold more abundant within BBD than in CP (data not shown). Previous experimental work has failed to demonstrate that the BBD-dominating cyanobacterium, *R. reptotaenium*, performs sulfide-dependent anoxygenic photosynthesis since a non-axenic culture of *R. reptotaenium* did not survive in the presence of dichlorophenyldimethyl urea (DCMU, an inhibitor of electron flow in PSII (Trebst 1980)) and sulfide under light conditions (Myers and Richardson 2009). However, it has also been observed that DCMU does not inhibit the experimentally-induced development of BBD or the progression of active BBD lesions dominated by *R. reptotaenium* on coral colonies (Richardson *et al.* 2009). Assuming that DCMU effectively inactivates PSII of *R. reptotaenium*, these results indicate two possibilities: (1) The cyanobacterium can grow heterotrophically, or (2) it performs photosynthesis independent of the oxygen-evolving PSII, *e.g.* anoxygenic photosynthesis using sulfide as an electron-donor. The former is not well-supported since a recent culture-based incubation experiment demonstrated that *R. reptotaenium* does not survive heterotrophically without light (Stanic *et al.* 2011), whereas the latter is more supported given the presence sulfide in deeper anoxic areas of the BBD lesion (Carlton and Richardson 1995, Glas *et al.* 2012) and the sequence-based evidence of sulfide-quinone reductase expression presented in this study. Furthermore, a field study observed sulfide oxidizing bacteria (SOB)-like organisms on the surface of the BBD lesion under high-light conditions, and it was proposed that the phenomenon was due to occasional non-oxygenation of the mat surface under light because these SOB are expected to be at the oxygen-sulfide interphase (Viehman and Richardson 2002). This field observation thus

provides additional support for the capability of the BBD cyanobacterium to perform anoxygenic photosynthesis without relying on PSII.

The *isiA* gene, coding a chlorophyll-binding protein IsiA, also appeared to be highly expressed in BBD (Table 2). This protein is induced in iron-deficient conditions and protects cells from light-induced damage by increasing the size of a photo-harvesting antenna attached to PSI, thereby converting light-energy to heat (Wilson *et al.* 2007). Iron-deficiency is especially critical for cyanobacteria as their photosynthetic apparatus requires between 22 and 23 molecules of iron per compound (Ferreira and Straus 1994). Cyanobacteria, like other Bacteria, adapt to iron-limiting environments by secreting iron-scavenging siderophores and substituting ferredoxin with flavodoxin (Ferreira and Straus 1994). In this study, Cya2-genes coding a siderophore transporter and flavodoxin were upregulated and 3 out of 4 [2Fe-2S] ferredoxins were downregulated in BBD relative to CP, although these differences were not statistically significant (data not shown). These observations suggest that the BBD cyanobacterium has strategies to mitigate the adverse-effects of iron-deficiency and grow at a high-density. Within the BBD lesion, cyanobacterial filaments are found to be neatly aligned (Miller *et al.* 2012), which may be responsible for intensifying vertical stratification of microenvironmental conditions in BBD lesions compared to CP lesions (Glas *et al.* 2012). These sequence-based results provide further support for the role of tightly-aggregated cyanobacterial biomass in BBD pathogenesis through retention of anoxic and sulfidic conditions in the BBD lesion.

Transcriptomic patterns of the gammaproteobacterial bin Oce

Profiles of transcriptomes mapped to the metagenomic bin Oce, sharing 98% sequence identity with the 5S rRNA gene sequence of *Thalassolituus oleivorans* R6-15

(GenBank Acc. CP006829; belonging to the order Oceanospirillales), indicated differential gene expression patterns between CP and BBD libraries in a wide range of functions (Suppl. Table 2). Sequence regions relatively more expressed in BBD than in CP included those coding fatty acid metabolism, glycerol metabolism, ATP synthesis and virulence factor, while sequences relatively more expressed in CP than in BBD included genes associated with virulence regulation, stress response, oxidative stress resistance, polysaccharide synthesis and secretion (Suppl. Table 2). Notably, isocitrate lyase (*icl*)- and aerobic glycerol-3-phosphate dehydrogenase (*glpD*)-coding genes were relatively more expressed in BBD than CP. Isocitrate lyase is an enzyme essential to the glyoxylate bypass, an anaplerotic route for replenishing the TCA cycle through assimilation of acetyl coenzyme A (Kornberg and Krebs 1957). Beta-oxidation of fatty acids produces substantial acetyl coenzyme A and therefore the glyoxylate bypass is important for heterotrophic growth of bacteria utilizing fatty acid substrates (Kornberg and Krebs 1957, McKinney *et al.* 2000). An aerobic glycerol-3-phosphate dehydrogenase is an essential enzyme for aerobic (*i.e.* oxygen-consuming) growth of bacteria utilizing glycerol (Cozzarelli *et al.* 1965). Therefore, greater expression of these enzymes in BBD together suggests that Oce bacteria more actively utilize lipids as growth substrates and consumes oxygen in BBD than in CP. Fatty acids utilized by Oce may be at least partially sourced from degraded cyanobacterial fatty acids, of which higher synthetic gene abundance and expression were observed in BBD than in CP (Figure 3; labelled as ‘mycolic acid synthesis’; also see the main document). Another possible source of lipids is degrading coral tissues since (1) rates of disease progression are faster in BBD than CP (Sato *et al.* 2010), providing more coral-derived organic matter to the BBD lesion-associated microbial communities (Sato *et al.* 2015), and (2) 10~30% of the dry weight of coral tissue consists of lipids, an important energy storage for the animal, in the form of phospholipids, diacylglycerol, free fatty acids, and triacylglycerol (Harland *et al.* 1993, Grottoli *et al.* 2004).

Furthermore, bacteria represented by Oce may perform robust energy production potentially with this increased lipid substrates in BBD. This is supported by significantly increased relative abundance of transcriptomes coding F0F1-type ATP synthase subunits in BBD (*atpA, atpA2, LOR_35c02990*; Suppl. Table 2), and 11 out of 13 other genomic regions coding subunits of F0F1-type ATP synthase were also more expressed in BBD than in CP (data not shown). Oce in CP displayed higher expressions of genes coding a transcriptional regulatory CpxR protein and RseC protein, both indicative of envelope stress responses (Missiakas *et al.* 1997, Bianchi and Baneyx 1999, Buelow and Raivio 2005), and an alkyl hydroperoxide reductase subunit F (*ahpF*) indicative of oxidative stress (Poole *et al.* 2000). These gene-expression patterns indicative of stress suggest that microenvironmental conditions within CP lesions are not preferred conditions for Oce. In addition, an increased expression of a gene in CP coding a RNA polymerase sigma-H factor (*algU*), an initiator of polysaccharide biosynthesis in the form of alginate (Martin *et al.* 1993), may indicate enhanced bacterial adaptation to oxidative stress by energy investment in mucoid surface production (Mathee *et al.* 1999, Sabra *et al.* 2002). Interestingly, Oce in BBD indicated a greater gene expression of a virulence factor (*sigL*) and a lower expression of a virulence down-regulator (*cdpA*) than within CP. Extracytoplasmic function sigma factor SigL has an important role in pathogenesis as it controls the pathogen’s persistence through surface interaction with infected host (Hahn *et al.* 2005, Dainese *et al.* 2006), whereas the downregulation of a 3’,5’-cyclic adenosine monophosphate phosphodiesterase CdpA elevates the intercellular concentration of cyclic adenosine monophosphate (cAMP), thereby enhancing the expression of a number of cAMP-dependent virulence genes such as exotoxin-, type 3 secretion system- and effector protein-coding genes (Fuchs *et al.* 2010). In summary, observed patterns in the mapped transcriptomes in Oce collectively suggest that (1) an increased influx of total lipids derived from degrading BBD-cyanobacteria and/or coral

tissues at the BBD stage enhances energy-generating metabolism of Oce bacteria, (2) the enhanced metabolism may confer further virulence to the Oce bacteria within BBD lesions, potentially further damaging coral tissue through host-microbial interactions, and (3) increased oxygen-consuming heterotrophy on lipids observed in BBD will further contribute to the formation of anoxia within the BBD lesion, one of the major virulent biogeochemical factors in BBD (Glas *et al.* 2012).

Given an indication of high genetic contamination in the Oce bin (Table 1), the above interpretations of the observed gene expression require extra caution as they may not be derived from a single species population. However, even if genomic sequences in Oce were derived from more than one bacterial species, biological indications inferred from the gene expression profiles of Oce-associated organism(s) provide insights into their role in the overall lesion functioning of the BBD-associated community as a collective taxonomic group. This is because Oce was assembled with sequences sharing similar taxonomic positions, coverage ratio in CP and BBD, and nucleotide signatures such as GC% and tetranucleotide-frequency. To resolve gene expression patterns of Oce at a single-species level, the examination of these hypotheses requires further investigations using more focused approaches, such as single-cell genomics.

Little sequence-based evidence for involvement of toxin-related genes in BBD pathogenesis

Pathogenicity of the BBD microbial mat has also been attributed to other microbiologically-mediated virulence factors including the biosynthesis of cyanotoxins (Richardson *et al.* 2007) and *Vibrio*-toxins (Barneah *et al.* 2007, Arotsker *et al.* 2009). Production of nodularin and microcystin has been demonstrated from BBD lesions and BBD-

derived cyanobacterial cultures from the Caribbean (Gantar *et al.* 2009, Stanic *et al.* 2011) although these toxins were not detected in BBD found on the GBR (Glas *et al.* 2010). We searched BBD and CP datasets against a set of publically-available nucleotide sequences that code biosynthesis pathways of nodularin (*ndaABCDEFGHI*) and microcystin (*mcyABCDEGJ*) using tBLASTx (Camacho *et al.* 2009; e-value $\leq 10^{-5}$). Sequences associated with nodularin-synthesizing genes were relatively more abundant in CP than BBD in both metagenomes and metatranscriptomes (Suppl. Figure 9), suggesting that nodularin is not linked to the increase of lesion-virulence during the onset of BBD from CP (Sato *et al.* 2010). The relative abundance of microcystin-synthesizing genes were 1.5-fold lower in the metagenome of BBD than that of CP, although the metatranscriptomes indicated that they were 1.2-times more highly represented in BBD than in CP (Suppl. Figure 9). Given that the relative sequence abundance of the Cya2 bin representing the BBD-dominating cyanobacteria was approximately 291-times higher in the BBD metagenome than CP metagenome (Table 1), it is unlikely that these microcystin synthesizing genes are harbored by the Cya2 cyanobacterium. Instead, the presence of other cyanobacterial types that represent minor constituents of BBD and CP lesions have been demonstrated in the GBR region (Sato *et al.* 2010, Sato *et al.* 2013), and thus these may be the host of the microcystin and nodularin biosynthesis sequences observed in the datasets. Gene-prediction and functional annotation of sequences within the BBD-dominating Cya2 genomic bin also indicated that microcystin production genes are not present in the Cya2 sequences. The Cya2 bin demonstrated a high genome coverage (99%; Table 1) and therefore the inability to observe microcystin production genes in Cya2 is unlikely due to missing gene reads or a lack of completeness in this bin, but more likely due to the region-specific variation of BBD-associated cyanobacterial genotypes among the Caribbean, Red Sea and Info-Pacific coral reefs (Richardson *et al.* 2007, Glas *et al.* 2010). One gene-coding region (7,056-nt) in Cya2 was

annotated as an amino acid adenylation enzyme/thioester reductase family protein and shared 48% amino acid sequence identity with a puwainaphycin-production gene *puwA*, which is responsible for the final peptidyl elongation prior to the cyclization of this cytotoxic lipopeptide (Mareš *et al.* 2014). This may indicate the presence of a potential cyanotoxin-associated gene sequence in BBD-dominating cyanobacterial sequences obtained from the GBR. However, transcriptomes that were mapped against this sequence represented extremely low proportions (0.002% and 0.003% of the total number of transcriptomes mapped against Cya2 in CP and BBD libraries, respectively), and the gene sequence arrangement in the Cya2 around this candidate *puwA* region does not indicate the domain structure required for the synthesis of puwainaphycin (Mareš *et al.* 2014). Therefore, its potential function and contribution to the overall BBD pathogenicity remain unclear and subject to further investigations. Our finding contrasts the previous metatranscriptomic study by Arotsker *et al.* (2016), which has proposed that the most transcribed gene in the BBD lesion, namely cyanobacterial adenosylhomocysteinase, is involved in cyanotoxin production. However, a link between the adenosylhomocysteinase and cyanotoxin production has not been established, as adenosylhomocysteinase is one of the most wide-spread, highly conserved proteins among prokaryotes and eukaryotes and plays a critical role in the metabolism of sulfur-containing amino acids (Sganga *et al.* 1992).

Metagenomic and metatranscriptomic sequences that were affiliated to the virulence factors of *Vibrio* spp. (*i.e.* all genes included in SEED classifications of '*Vibrio* pathogenicity island' and 'Cytolysin and lipase operon in *Vibrio*') were also compared between CP and BBD datasets. While metagenomic sequences coding *Vibrio*-virulence factors were relatively more abundant in BBD than in CP, those in metatranscriptomic datasets were more abundant in CP than BBD (Suppl. Figure 9). These results suggest that even though functional genes

associated with the pathogenicity of *Vibrio* were present in BBD metagenomes, those sequences are not actively transcribed in the BBD lesion compared to the CP lesion. Sequences associated with *Vibrio* zinc-metalloproteases, a known gene associated with coral tissue lysis (Sussman *et al.* 2008, Sussman *et al.* 2009), were also only confirmed at low relative abundance within the CP-metagenome and the BBD-transcriptomes (representing only 0.0005% and 0.00003% of functionally annotated sequences in each dataset, respectively). Together, these observations suggest that *Vibrio*-related toxins are probably not a major factor involved in the development of BBD pathogenicity during the onset of disease originated from CP, and thus *Vibrio* spp. within the BBD lesions are likely secondary opportunists among other heterotrophic organisms utilizing available organic substrates (Saeed 1995, Wendling and Wegner 2013).

2. Supplementary method

Protocols for microbial messenger RNA enrichment from coral disease lesion samples

Workflow

1. Synthesizing rRNA probes
    1.1. PCR-amplification of SSU and LSU rRNA coding regions from DNA samples
    1.2. *In vitro* transcription (IVT) of PCR products with biotin-labelled nucleotides
    1.3. DNAse-digest and purification of rRNA probes

2. Hybridization and subtraction of rRNA
    2.1. Hybridization of total RNA with biotinylated rRNA probes
    2.2. Subtraction of hybridized rRNA
    2.3. Clean up remaining RNA samples
    2.4. Subtraction of poly(A)-tailed mRNA
    2.5. Clean-up and concentration of mRNA-enriched samples
    2.6. Quantification and quality-check of mRNA-enriched samples

3. Amplification of mRNA-enriched samples
    3.1. Amplification of mRNA-enriched RNA samples with T7-BpmI-d(T)16-VN primer
    3.2. Quantification of amplified RNA samples and concentration-adjustment

4. Double-stranded cDNA synthesis
    4.1. First-strand cDNA synthesis with random primer
    4.2. Second-strand cDNA synthesis
    4.3. Quantification of double-stranded cDNA
    4.4. *BpmI*-digestion
    4.5. Purification of digested cDNA
    4.6. Quantification of clean digested cDNA
    4.7. Visualize cDNA size distributions

Consumables required

1. Synthesizing rRNA probes

   PCR primers targeting ribosomal SSU or LSU with T7 promoter sequence for Bacteria, Archaea and Eukaryota (Stewart *et al.* 2010).

   BioXact Short DNA Polymerase kit (Bioline, London, BIO-21065)

   NucleoSpin Gel and PCR Clean-up (Machery-Nagel, Düren, 740609)

   MEGAscript T7 Kit (Ambion, Austin, AM1333)

   SUPERase-In RNase inhibitor (Ambion, AM2694)

   Biotin-11-CTP and Biotin-16-UTP (Enzo Life Science, Farmingdale, NY, ENZ-42818 & 42814)

   MEGAclear Kit (Ambion, AM1908)

2. Hybridization and subtraction of rRNA

   20X Sodium chloride citrate (SSC) buffer (Ambion, AM9770)

   Formamide 100% (Sigma-Aldrich, St. Louis, MI)

   Streptavidin-coated magnetic beads (New England BioLabs, Ipswich, MA, S1420S)

   Oligo d(T)25-coated magnetic beads (New England BioLabs, S1419S)

   SUPERase-In RNase inhibitor (Ambion, as above)

   RNeasy MinElute Cleanup kit (Qiagen, Hilden, 74204)

   2100 Bioanalyzer RNA 6000 Pico chip kit (Agilent, Santa Clara, CA, 5067-1513)

   0.1M NaOH solution

3. Amplification of enriched mRNA

   MessageAmp II Bacteria kit (Ambion, AM1790)

   T7-BpmI-dT16VN primer (Stewart *et al.* 2010)

   SUPERase-In RNase inhibitor (Ambion, as above)

   Super Script III First Stranded Synthesize System for RT-PCR (Invitrogen, 18080-051)

4. Double-stranded cDNA synthesis

   Contents in Super Script Second Stranded Synthesize kit (Invitrogen, 11917-010):

   - Second Strand Reaction Buffer 5x (Invitrogen, 18080-400)
   - dNTP 2.5mM ea. mix (Bioline, BIO-39044)
   - E. coli DNA ligase (Invitrogen, 18052-019)
   - E. coli DNA Polymerase I (Invitrogen, 18010-017)

T4 DNA Polymerase (Invitrogen, 18005-017)
E. coli Ribonuclease H (RNase H, Invitrogen, 18021-014)
NucleoSpin Gel and PCR Clean-up (Machery-Nagel, as above)
BpmI restriction enzyme (New England BioLabs, R0565S)
Qubit dsDNA HS Assay Kit (Invitrogen, Q32851)

Protocols

1. Synthesizing rRNA probes
   1.1. PCR-amplification of bacterial, archaeal and eukaryotic SSU and LSU rRNA coding genes with primers including a T7 promoter region and clean-up of the PCR products; yielding 6 different PCR-products per DNA sample.
   - Follow PCR protocol from Stewart *et al.* (2010) in Supplementary Material with the following conditions and DNA samples (derived from the same microbial lesions as the one used for the extraction of target total RNA):

Reaction	10x Buffer (BioXact)	5 μl
	dNTP mix (2.5 mM ea.)	5 μl
	Hi-Spec Solution (BioXact)	2.5 μl
	MgCl2 (50 mM)	1 μl<br>(2 μl for Archaeal SSU-PCR)
	Forward/reverse primers	1.25 μl ea.
	BioXact Polymerase	0.5 μl
	Water and template	to 50 μl

Cycles 95°C x 5 min
35 cycles of
95°C x 20 sec
55°C x 20 sec (39°C for Bacterial LSU)
72°C x 2 min
72°C x 3 min

- Clean up PCR products with NucleoSpin Gel and PCR Clean-up kit and measure DNA-concentration with NanoDrop 2000 (Thermo Fisher Scientific,

Waltham, MA, USA). Adjust the concentration to 250~500 ng/µl with Elusion Solution (NucleoSpin Gel and PCR Clean-up kit) in 10 – 30 µl. PCR products can be pooled before clean-up if required.

1.2. *In-vitro* transcription (IVT) of PCR products with biotin-labelled CTP and UTP

- Follow IVT protocol from Stewart *et al.* (2010) in their Supplementary Material using MEGA script kit with a 14 hr IVT-incubation time. Each reaction should yield approximately 1 µg/µl of RNA probe for 500 ng of template PCR product.

1.3. DNAse-digest (TURBO DNAse, included in the IVT kit) and purification using MEGAclear kit.

- After TURBO DNAse treatment and purification using MEGAclear kit, elute RNA probes in 100 µl of Elution Solution (Follow the elution option-2 in MEGAclear kit manual).

2. Hybridization and subtraction of rRNA (mRNA enrichment)

2.1. Hybridization of total RNA and biotinylated rRNA probes, and subtraction of hybridized rRNA with streptavidin-coated magnet beads.

- Protocols were based on Stewart *et al.* (2010) as describes in Supplementary Material with the following modifications: Using 6 different types of rRNA probes per total RNA sample, total abundance of probes to template total RNA ratios was set 2:1 in RNA-weight. Composition of bacterial, archaeal and eukaryotic rRNA were respectively estimated as 76%, 5% and 19% from results of metagenomic taxonomic profiling, and the equal amount in weight of SSU and LSU-targeted probes were added within the same domain. In the clean-up step of subtracted RNA using RNeasy MinElute Cleanup kit, elute in 26 µL water (resulting in the final volume of 24 µL due to the dead space in elution-filter).

2.2. Subtraction of poly A-tailed mRNA with oligo(dT)-coated magnetic beads.

2.2.1. Wash oligo(dT) beads solution (50* µl x sample number, which can be pooled in a single 1.5 ml tube) once with the equal volume of 0.1 N NaOH and twice with the same volume of Biding Buffer**. Resuspend washed beads with a half

volume of Biding Buffer and divide into aliquots of 25* μL each in 1.5 mL tubes and keep them on ice.

* This should remove maximum of 500 ng of polyA mRNA. Increase all volumes proportionally if required.

**Biding Buffer contains; 20 mM Tris-HCl (pH7.5), 1.0 M LiCl, 2 mM EDTA (Filter-sterilized through a 0.22 μm, stored at 4 °C).

2.2.2. Adjust rRNA-subtracted RNA samples to 25* μl each in a 200 μL PCR tubes, by adding 1 μl of SUPERase-IN RNase inhibitor (and water if required) and add 25* μl of Binding Buffer.

2.2.3. Heat-denature RNA sample mixture at 65°C for 2 min, then immediately chill on ice.

2.2.4. Add RNA sample to the washed beads suspended in Binding Buffer above.

2.2.5. Hybridise RNA sample and beads at room temperature for 5min in continuous inverting.

2.2.6. Capture beads on a magnet stand for 2 min and transfer the supernatant containing subtracted RNA (approximately 75* μl) in a new 1.5 ml tube.

2.2.7. Resuspend the remaining beads with 50* μl of Washing Buffer***.

***Washing Buffer contains; 10 mM Tris-HCl (pH7.5), 0.15 M LiCl, 1 mM EDTA

(Filter-sterilized through a 0.22 μm, stored at 4 °C).

2.2.8. Capture beads on a magnet stand for 2 min, and transfer the supernatant (approximately 50* μl) in the above 1.5 ml tube containing subtracted RNA (mRNA-enriched sample is now in total volume of 125* μl each).

2.3. Clean and concentrate with RNeasy MinElute Cleanup kit.

- Elute in 13.5 μL of water (resulting in the final volume of approximately 11.5 μL).

2.4. Check RNA contents with 2100 Bioanalyzer and RNA 6000 Pico chip kit.

- Measure 1.5 μL of enriched RNA sample by following the kit-instructions, but do not use dilution series as concentrations may be very low. Verify that distinct peaks indicating SSU and LSU rRNA are substantially reduced.

3. Amplification of enriched mRNA and cDNA synthesis

3.1. Amplification of mRNA-enriched RNA samples with MessageAmp II Bacteria kit using T7-BpmI-d(T)16-VN primer.

- Follow the manual of MessageAmp II Bacteria kit with the following modifications and notes:
    - Use up to 6.5 μl of template RNA (approximately 20 ng).
    - In the initial heat-denaturing step (Step C2), reduce 70°C incubation time to 5 min.
    - Before making the polyadenylation master mix (Step C3), vortex the 10X Poly(A) Tailing Buffer very well to dissolve precipitation that often forms in freezer. If 6.5 μL of RNA sample is used, add no water and transfer 3.5 μL of master mix to RNA sample.
    - In the reverse transcription master mix (Step D1), replace T7-Oligo(dT)-VN primer with 1 μL of the modified T7-BpmI-d(T)16-VN primer (Stewart *et al.*, 2010) at the concentration of 10 μM. Before making the master mix, vortex the First Strand Buffer very well to dissolve precipitation often formed in the freezer.
    - Before proceeding to the second strand cDNA synthesis (Step E1), pre-chill thermal cycler to 16°C.
    - In the washing step during cDNA purification (Step F4c), spin with sample-tubes' lid open.
    - In the *in vitro* transcription (Step G1), add 1 μl of SUPERase-In RNase inhibitor in the master mix (optional; 25 μl in total). Also, vortex the T7 Reaction Buffer very well to dissolve precipitation that easily forms in the freezer. In Step G3, add 59 μl of water to make the total volume 100 μl.
    - In the elution step during aRNA purification (Steps H6) add 52 μl of water to the filter to yield higher concentrations, and incubate at 55°C for 10 min.

3.2. Quantification of amplified mRNA enriched samples with NanoDrop2000, and concentrate to 0.8~1.0 μg/μl with vacuum drier (without heating) if required.

4. Double-stranded cDNA synthesis

4.1. First-strand cDNA synthesis using random hexamers with SuperScript III First Strand Synthesis System.

- Follow the kit manual up to the synthesis of cDNA and termination of the reaction (until Step 6), with the following modifications:
  - In the reaction component, double the volume of Random Hexamer Primers and Superscript III RT (Steps 2 and 4). Duplicate reactions per RNA sample using up to 5 μg of template RNA each (*i.e.* total template RNA is up to 10 μg per sample).
  - In Step 2, add the total of RNA sample and water up to 6 μL (*i.e.* the total volume of reaction is 9 μL, including 2 μL of Rabdin hexamers and 1 μL of dNTP mix).
  - In Step 4, total volume of master mix is 11 μL per reaction (increase each component by 5% in the master mix for pipetting loss). Ensure to follow the order of reactions to be mixed.
  - After Step 6, pool duplicate reactions (using the same RNA template) in a new 600 μL tube on ice.

4.2. Second-strand cDNA synthesis based on SuperScript Double Stranded cDNA kit

- Follow the manual from Page 3 (Second-Strand Synthesis), until the incubation with T4 DNA Polymerase incubation (Step 3) with the following modifications and notes:
  - Double all the reaction components.
  - While mixing the reaction (Step 1), ensure to follow the order of reagents shown on the manual and add each to the first strand cDNA reaction (*i.e.* do not make a master mix). If 2.5 mM ea. dNTP mix (instead of 10 mM ea. dNTP mix shown in manual) is used, add 24 μL of dNTP mix and reduce water to 164 μL. Gently mix and split the reaction to three of 100 μl reactions in 200 μL PCR tubes on ice. Before proceeding to Step 2, pre-chill the thermal cycler to 16°C.
  - Step 2; incubate for 2.5 hrs at 16°C.
  - Step 3; add 1.33 μl of T4 DNA Polymerase in each tube and mix gently, and incubate the reaction for 15 mins at 16°C.

- After Step 3, purify double-stranded cDNA with NucleoSpin Gel and PCR Clean-up Kit and elute cDNA in 42 µl of Elution Solution.

4.3. Quantify cDNA with Qubit dsDNA HS kit using NanoDrop3300 (Thermo Fisher Scientific, Waltham, MA, USA) by following the manuals.

4.4. BpmI-digestion of double-stranded cDNA to remove poly-A tails (modified from Stewart *et al.*, 2010).

- Mix the following reaction for digestion in a 1.5 mL tube;

Double stranded cDNA	40 µl
10x NE Buffer 3	5.4 µl
100x BSA	0.6 µl
BpmI	8.0 µl

- Incubate at 37°C for 1.5 hrs. After the incubation, add another 4 µl of BpmI and incubate further 1.5 hrs at 37°C, followed by the heat inactivation at 70°C for 20 min.

4.5. Purification of digested cDNA.

- Use NucleoSpin Gel and PCR Clean-up Kit with the following modification:
  - At the final step, elute cDNA with 30 µl of Elution Solution first, and elute again with another 36 µl of Elution Solution.

4.6. Quantification of clean digested cDNA (as step 4.3).

4.7. Visualize cDNA size distributions by the electrophoresis of 5 µl cDNA on 1% agarose gel with ethidium bromide staining.

References

Arieli B, Shahak Y, Taglicht D, Hauska G, Padan E. (1994). Purification and characterization of sulfide-quinone reductase, a novel enzyme driving anoxygenic photosynthesis in *Oscillatoria limnetica*. *Journal of Biological Chemistry* **269:** 5705-5711.

Arotsker L, Siboni N, Ben-Dov E, Kramarsky-Winter E, Loya Y, Kushmaro A. (2009). *Vibrio* sp. as a potentially important member of the Black Band Disease (BBD) consortium in *Favia* sp. corals. *FEMS Microbiology Ecology* **70:** 183-192.

Arotsker L, Kramarsky-Winter E, Ben-Dov E, Kushmaro A. (2016). Microbial transcriptome profiling of black band disease in a *Faviid* coral during a seasonal disease peak. *Diseases of Aquatic Organisms* **118:** 77-89.

Atad I, Zvuloni A, Loya Y, Rosenberg E. (2012). Phage therapy of the white plague-like disease of *Favia favus* in the Red Sea. *Coral Reefs* **31:** 665-670.

Barneah O, Ben-Dov E, Kramarsky-Winter E, Kushmaro A. (2007). Characterization of black band disease in Red Sea stony corals. *Environmental Microbiology* **9:** 1995-2006.

Bianchi AA, Baneyx F. (1999). Hyperosmotic shock induces the σ32 and σE stress regulons of *Escherichia coli*. *Molecular Microbiology* **34:** 1029-1038.

Buelow DR, Raivio TL. (2005). Cpx signal transduction is influenced by a conserved N-terminal domain in the novel inhibitor CpxP and the periplasmic protease DegP. *Journal of Bacteriology* **187:** 6622-6630.

Camacho C, Coulouris G, Avagyan V, Ma N, Papadopoulos J, Bealer K *et al*. (2009). BLAST+: architecture and applications. *BMC Bioinformatics* **10:** 421.

Carlton RG, Richardson LL. (1995). Oxygen and sulfide dynamics in a horizontally migrating cyanobacterial mat - Black band disease of corals. *FEMS Microbiology Ecology* **18:** 155-162.

Chénard C, Suttle CA. (2008). Phylogenetic diversity of sequences of cyanophage photosynthetic gene psbA in marine and freshwaters. *Applied and Environmental Microbiology* **74:** 5317-5324.

Cohen Y, Jorgensen BB, Revsbech NP, Poplawski R. (1986). Adaptation to hydrogen-suflide of oxygenic and anoxygenic photosynthesis among cyanobacteria. *Applied and Environmental Microbiology* **51:** 398-407.

Cohen Y, Pollock JF, Rosenberg E, Bourne DG. (2013). Phage therapy treatment of the coral pathogen Vibrio coralliilyticus. *MicrobiologyOpen* **2:** 64-74.

Correa AMS, Ainsworth TD, Rosales SM, Thurber AR, Butler CR, Vega Thurber RL. (2016). Viral outbreak in corals associated with an in situ bleaching event: atypical herpes-like viruses and a new megavirus infecting Symbiodinium. *Frontiers in Microbiology* **7**.

Cozzarelli NR, Koch JP, Hayashi S, Lin EC. (1965). Growth stasis by accumulated L-alpha-glycerophosphate in *Escherichia coli*. *Journal of Bacteriology* **90:** 1325-1329.

Dainese E, Rodrigue S, Delogu G, Provvedi R, Laflamme L, Brzezinski R *et al*. (2006). Posttranslational regulation of *Mycobacterium tuberculosis* extracytoplasmic-function sigma factor σL and roles in virulence and in global regulation of gene expression. *Infection and Immunity* **74:** 2457-2461.

Ferreira F, Straus N. (1994). Iron deprivation in cyanobacteria. *Journal of Applied Phycology* **6:** 199-210.

Fuchs EL, Brutinel ED, Klem ER, Fehr AR, Yahr TL, Wolfgang MC. (2010). *In vitro* and *in vivo* characterization of the *Pseudomonas aeruginosa* cyclic AMP (cAMP) phosphodiesterase cpdA, required for cAMP homeostasis and virulence factor regulation. *Journal of Bacteriology* **192:** 2779-2790.

Gantar M, Sekar R, Richardson L. (2009). Cyanotoxins from black band disease of corals and from other coral reef environments. *Microbial Ecology* **58:** 856-864.

Glas MS, Motti CA, Negri A, Sato Y, Froscio S, Humpage AR *et al.* (2010). Cyanotoxins are not implicated in the etiology of coral black band disease outbreaks on Pelorus Island, Great Barrier Reef. *FEMS Microbiology Ecology* **73:** 43-54.

Glas MS, Sato Y, Ulstrup KE, Bourne DG. (2012). Biogeochemical conditions determine virulence of black band disease in corals. *ISME Journal* **6:** 1526-1534.

Grottoli AG, Rodrigues LJ, Juarez C. (2004). Lipids and stable carbon isotopes in two species of Hawaiian corals, *Porites compressa* and *Montipora verrucosa*, following a bleaching event. *Marine Biology* **145:** 621-631.

Hahn MY, Raman S, Anaya M, Husson RN. (2005). The *Mycobacterium tuberculosis* extracytoplasmic-function sigma factor SigL regulates polyketide synthases and secreted or membrane proteins and is required for virulence. *Journal of Bacteriology* **187:** 7062-7071.

Harland AD, Navarro JC, Spencer Davies P, Fixter LM. (1993). Lipids of some Caribbean and Red Sea corals: total lipid, wax esters, triglycerides and fatty acids. *Marine Biology* **117:** 113-117.

Holmfeldt K, Solonenko N, Shah M, Corrier K, Riemann L, VerBerkmoes NC *et al*. (2013). Twelve previously unknown phage genera are ubiquitous in global oceans. *Proceedings of the National Academy of Sciences* **110:** 12798-12803.

Kornberg HL, Krebs HA. (1957). Synthesis of cell constituents from C2-units by a modified tricarboxylic acid cycle. *Nature* **179:** 988-991.

Lindell D, Sullivan MB, Johnson ZI, Tolonen AC, Rohwer F, Chisholm SW. (2004). Transfer of photosynthesis genes to and from *Prochlorococcus* viruses. *Proceedings of the National Academy of Sciences of the United States of America* **101:** 11013-11018.

Mareš J, Hájek J, Urajová P, Kopecký J, Hrouzek P. (2014). A hybrid non-ribosomal peptide/polyketide synthetase containing fatty-acyl ligase (FAAL) synthesizes the β-amino fatty acid lipopeptides puwainaphycins in the Cyanobacterium Cylindrospermum alatosporum. *PLoS ONE* **9:** e111904.

Martin DW, Holloway BW, Deretic V. (1993). Characterization of a locus determining the mucoid status of *Pseudomonas aeruginosa*: AlgU shows sequence similarities with a *Bacillus* sigma factor. *Journal of Bacteriology* **175:** 1153-1164.

Mathee K, Ciofu O, Sternberg C, Lindum PW, Campbell JIA, Jensen P *et al*. (1999). Mucoid conversion of *Pseudomonas aeruginosa* by hydrogen peroxide: A mechanism for virulence activation in the cystic fibrosis lung. *Microbiology* **145:** 1349-1357.

McKinney JD, zu Bentrup KH, Munoz-Elias EJ, Miczak A, Chen B, Chan W-T *et al*. (2000). Persistence of *Mycobacterium tuberculosis* in macrophages and mice requires the glyoxylate shunt enzyme isocitrate lyase. *Nature* **406:** 735-738.

Miller AW, Blackwelder P, Al-Sayegh H, Richardson LL. (2012). Insights into migration and development of coral black band disease based on fine structure analysis. *Revista de Biologia Tropical* **60:** 21-27.

Miller SR, Bebout BM. (2004). Variation in sulfide tolerance of photosystem II in phylogenetically diverse cyanobacteria from sulfidic habitats. *Applied and Environmental Microbiology* **70:** 736-744.

Missiakas D, Mayer MP, Lemaire M, Georgopoulos C, Raina S. (1997). Modulation of the *Escherichia coli* σE (RpoE) heat-shock transcription-factor activity by the RseA, RseB and RseC proteins. *Molecular Microbiology* **24:** 355-371.

Myers JL, Sekar R, Richardson LL. (2007). Molecular detection and ecological significance of the cyanobacterial genera *Geitlerinema* and *Leptolyngbya* in black band disease of corals. *Applied and Environmental Microbiology* **73:** 5173-5182.

Myers JL, Richardson LL. (2009). Adaptation of cyanobacteria to the sulfide-rich microenvironment of black band disease of coral. *FEMS Microbiology Ecology* **67:** 242-251.

Pollock F, Wood-Charlson E, van Oppen M, Bourne D, Willis B, Weynberg K. (2014). Abundance and morphology of virus-like particles associated with the coral *Acropora hyacinthus* differ between healthy and white syndrome-infected states. *Marine Ecology Progress Series* **510:** 39-43.

Poole LB, Reynolds CM, Wood ZA, Karplus PA, Ellis HR, Li Calzi M. (2000). AhpF and other NADH:peroxiredoxin oxidoreductases, homologues of low Mr thioredoxin reductase. *European Journal of Biochemistry* **267:** 6126-6133.

Price MN, Dehal PS, Arkin AP. (2009). FastTree: Computing large minimum evolution trees with profiles instead of a distance matrix. *Molecular Biology and Evolution* **26:** 1641-1650.

Pruesse E, Quast C, Knittel K, Fuchs BM, Ludwig W, Peplies J *et al*. (2007). SILVA: A comprehensive online resource for quality checked and aligned ribosomal RNA sequence data compatible with ARB. *Nucleic Acids Research* **35:** 7188-7196.

Richardson LL, Sekar R, Myers JL, Gantar M, Voss JD, Kaczmarsky L *et al*. (2007). The presence of the cyanobacterial toxin microcystin in black band disease of corals. *Fems Microbiology Letters* **272:** 182-187.

Richardson LL, Miller AW, Broderick E, Kaczmarsky L, Gantar M, Stanic D *et al*. (2009). Sulfide, microcystin, and the etiology of black band disease. *Diseases of Aquatic Organisms* **87:** 79-90.

Rinke C, Schwientek P, Sczyrba A, Ivanova NN, Anderson IJ, Cheng JF *et al*. (2013). Insights into the phylogeny and coding potential of microbial dark matter. *Nature* **499:** 431-437.

Sabra W, Kim EJ, Zeng AP. (2002). Physiological responses of *Pseudomonas aeruginosa* PAO1 to oxidative stress in controlled microaerobic and aerobic cultures. *Microbiology* **148:** 3195-3202.

Saeed MO. (1995). Association of *Vibrio harveyi* with mortalities in cultured marine fish in Kuwait. *Aquaculture* **136:** 21-29.

Sato Y, Willis BL, Bourne DG. (2010). Successional changes in bacterial communities during the development of black band disease on the reef coral, *Montipora hispida*. *ISME Journal* **4:** 203-214.

Sato Y, Willis BL, Bourne DG. (2013). Pyrosequencing-based profiling of archaeal and bacterial 16S rRNA genes identifies a novel archaeon associated with black band disease in corals. *Environmental Microbiology* **15:** 2994-3007.

Sato Y, Civiello M, Bell SC, Willis BL, Bourne DG. (2015). Integrated approach to understanding the onset and pathogenesis of black band disease in corals. *Environ Microbiol***:** DOI: 10.1111/1462-2920.13122.

Sganga MW, Aksamit RR, Cantoni GL, Bauer CE. (1992). Mutational and nucleotide sequence analysis of S-adenosyl-L-homocysteine hydrolase from *Rhodobacter capsulatus*. *Proceedings of the National Academy of Sciences of the United States of America* **89:** 6328-6332.

Soffer N, Brandt ME, Correa AMS, Smith TB, Thurber RV. (2014). Potential role of viruses in white plague coral disease. *The ISME Journal* **8:** 271-283.

Stanic D, Oehrle S, Gantar M, Richardson LL. (2011). Microcystin production and ecological physiology of Caribbean black band disease cyanobacteria. *Environmental Microbiology* **13:** 900-910.

Stewart FJ, Ottesen EA, DeLong EF. (2010). Development and quantitative analyses of a universal rRNA-subtraction protocol for microbial metatranscriptomics. *ISME Journal* **4:** 896-907.

Sullivan MB, Lindell D, Lee JA, Thompson LR, Bielawski JP, Chisholm SW. (2006). Prevalence and evolution of core photosystem II genes in marine cyanobacterial viruses and their hosts. *PLoS Biology* **4:** 1344-1357.

Sussman M, Willis BL, Victor S, Bourne DG. (2008). Coral pathogens identified for white syndrome (WS) epizootics in the Indo-Pacific. *PLos ONE* **3:** e2393.

Sussman M, Mieog JC, Doyle J, Victor S, Willis BL, Bourne DG. (2009). *Vibrio* zinc-metalloprotease causes photoinactivation of coral endosymbionts and coral tissue lesions. *PLoS ONE* **4:** e4511.

Trebst A. (1980). Inhibitors in electron flow: Tools for the functional and structural localization of carriers and energy conservation sites. In: Anthony San P (ed). *Methods in Enzymology*. Academic Press. pp 675-715.

Viehman TS, Richardson LL. (2002). Motility patterns of *Beggiatoa* and *Phormidium corallyticum* in black band disease. *Proceedings of the ninth international coral reef symposium* **2:** 1251-1255.

Wendling CC, Wegner KM. (2013). Relative contribution of reproductive investment, thermal stress and *Vibrio* infection to summer mortality phenomena in Pacific oysters. *Aquaculture* **412-413:** 88-96.

Weynberg KD, Wood-Charlson EM, Suttle CA, van Oppen MJH. (2014). Generating viral metagenomes from the coral holobiont. *Frontiers in Microbiology* **5**.

Weynberg KD, Voolstra CR, Neave MJ, Buerger P, van Oppen MJH. (2015). From cholera to corals: Viruses as drivers of virulence in a major coral bacterial pathogen. *Scientific Reports* **5:** 17889.

Wilson A, Boulay C, Wilde A, Kerfeld CA, Kirilovsky D. (2007). Light-induced energy dissipation in iron-starved cyanobacteria: Roles of OCP and IsiA proteins. *The Plant Cell* **19:** 656-672.

Wood-Charlson EM, Weynberg KD, Suttle CA, Roux S, van Oppen MJH. (2015). Metagenomic characterisation of viral communities in corals: Mining biological signal from methodological noise. *Environmental Microbiology Reports***:** doi: 10.1111/1758-2229.12275.

3. Supplementary figures and tables

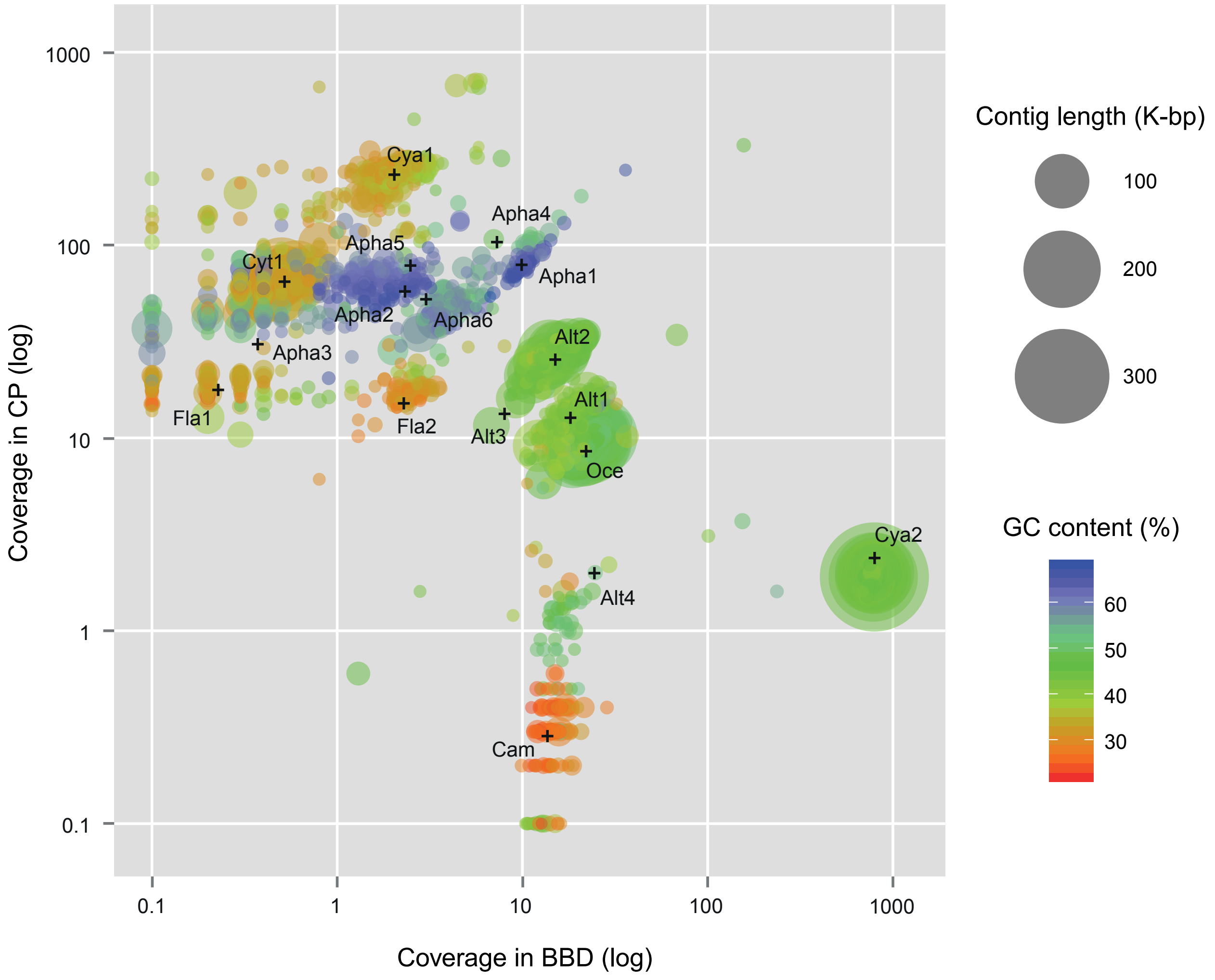

Supplementary Figure 1 Differential coverage and GC-content of metagenomic assembly recovered from microbial lesions of cyanobacterial patches (CP) and black band disease (BBD). Contigs larger than 5,000-bp with more than 0.1 mean coverage in both CP and BBD metagenomes are shown. Crosses indicate the mean coverage of the resulting genomic bins shown in Table 1.

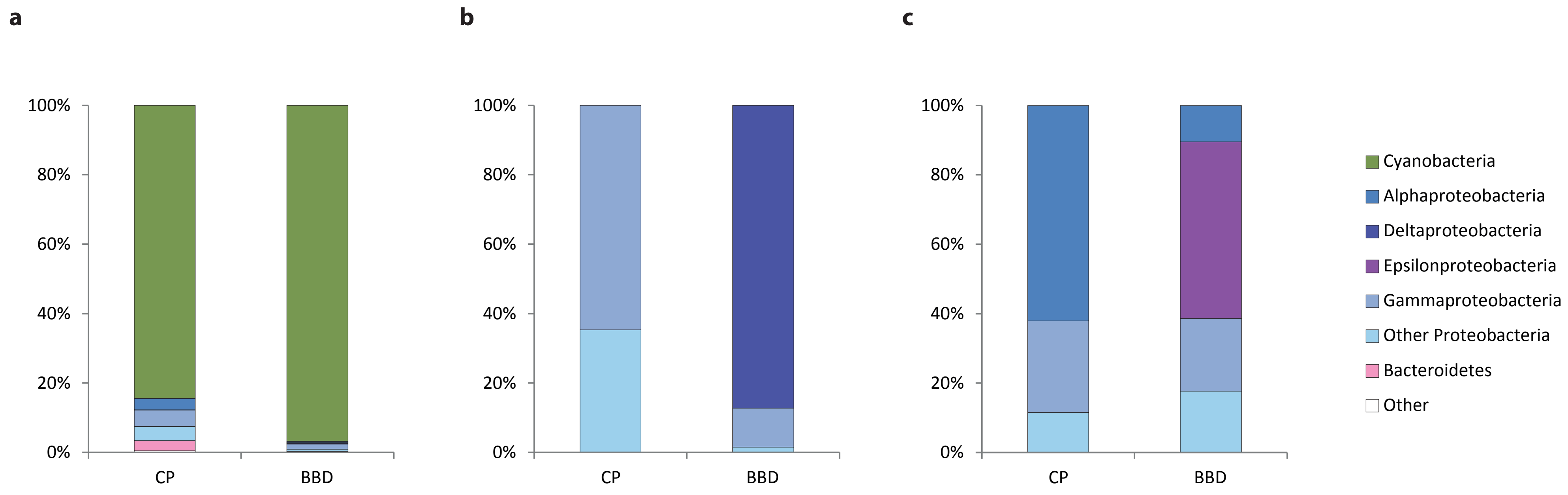

Supplementary Figure 2 Compositions of taxonomically assigned metatranscriptomic sequences associated with genes involved in (**a**) CO_2 fixation, (**b**) sulfate-reduction, and (**c**) sulfide-oxidation that were retrieved from microbial lesion samples of cyanobacterial patches (CP) and black band disease (BBD).

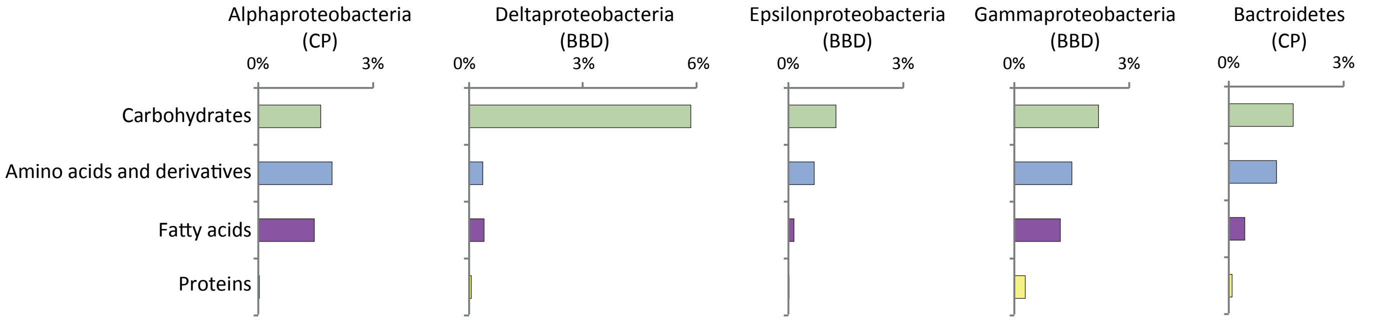

Supplementary Figure 3 Relative abundance of metatranscriptomic sequences associated with degradation and utilization of organic compounds within sequences of the major non-photosynthesizing taxonomic groups occurring in microbial lesions of cyanobacterial patches (CP) and black band disease (BBD). Metatranscriptomic sequences affiliated to each of the major taxonomic groups were first extracted from the dataset of CP or BBD, in which the corresponding taxonomic group represents a major component (shown in brackets; see Figure 2a). Proportions are calculated as the relative abundance of sequences within the total number of sequences that were assigned to all SEED Subsystems terms in each of the taxonomic group. SEED function terms that were included in the compound categories are listed in Supplementary Data 3.

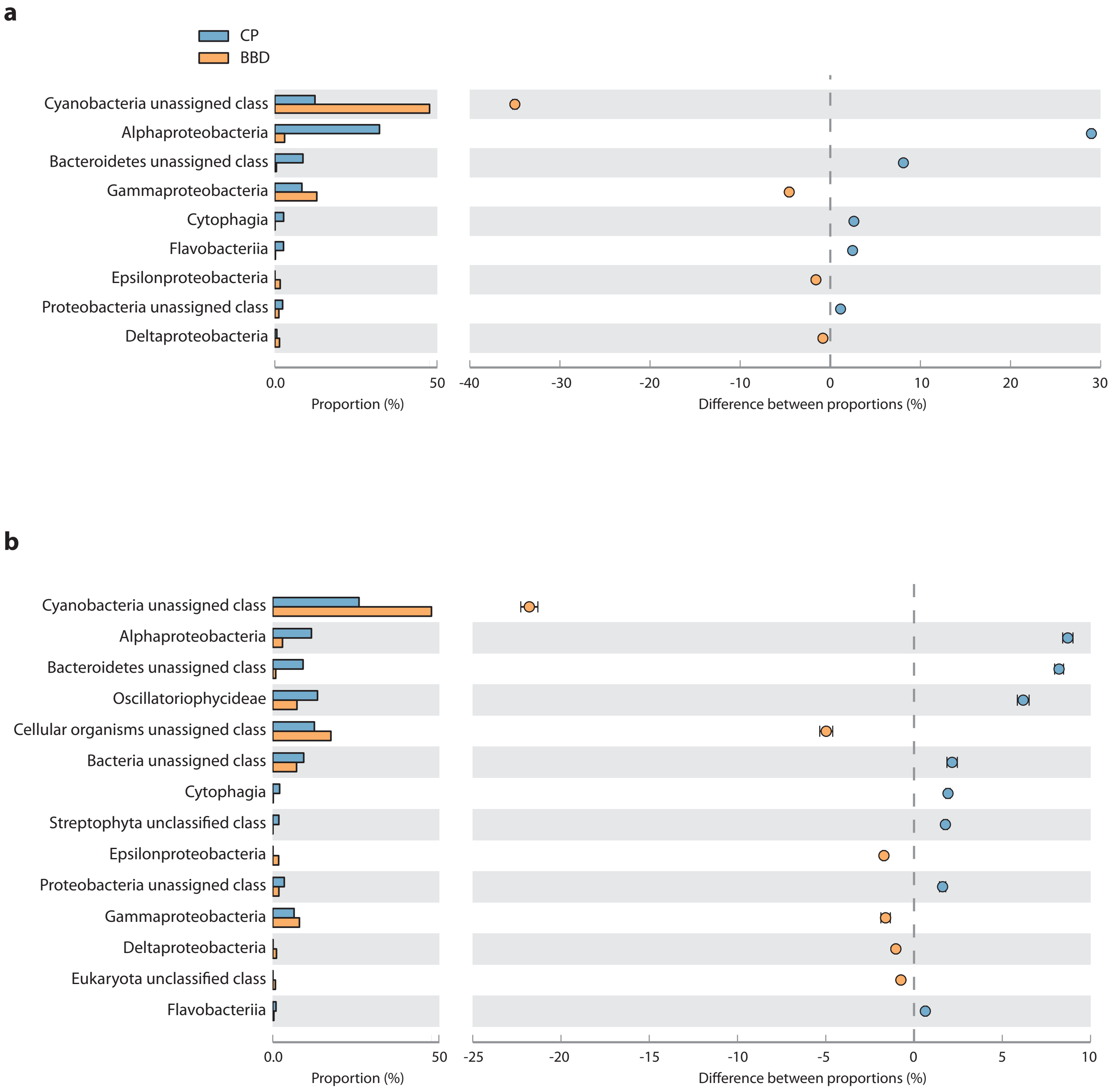

Supplementary Figure 4 Statistical comparisons of taxonomically annotated bacterial sequences at the class level in the (**a**) metagenomes and (**b**) metatranscriptomes derived from microbial lesions of cyanobacterial patches (CP) and black band disease (BBD). All proportions shown are calculated as the relative abundance of sequences within the indicated taxa divided by the total number of sequences that were matched with Universally Conserved Protein sequences (E-value < 1.0E-5; left, relative proportions; right, differences between proportions (negative value indicates BBD > CP) with 99.9% confidence intervals shown with error bars (most of them are not visible due to small ranges)). All differences in proportions were statistically significant (p-value < 1e-100).

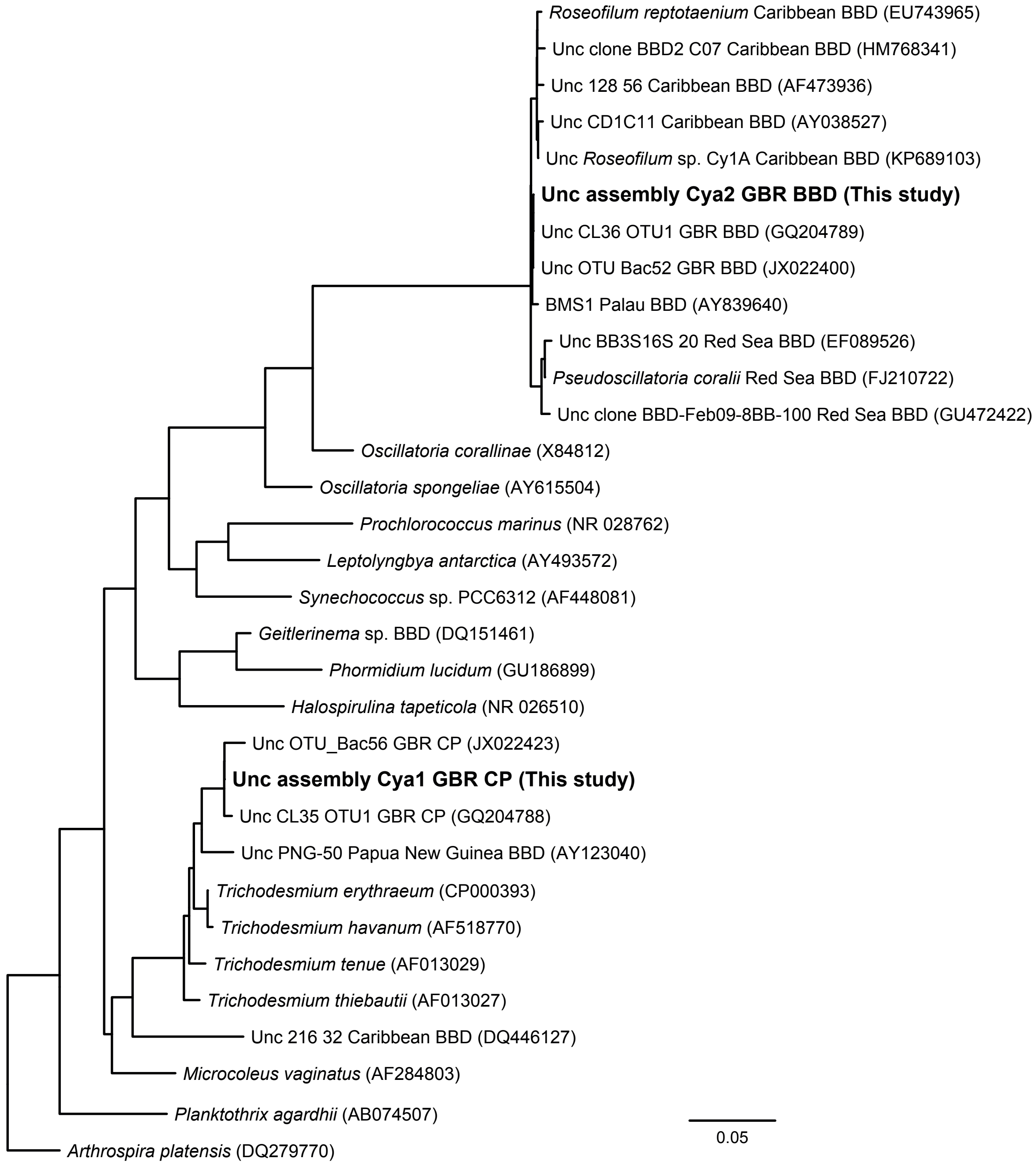

Supplementary Figure 5 Phylogeny of cyanobacterial 16S rRNA gene sequences recovered from microbial lesions associated with cyanobacterial patches (labelled as 'CP') and black band disease ('BBD') in relation to representative reference cyanobacterial sequences, including sequences retrieved from uncultured environmental samples (labelled as 'Unc'). Scale bar represents a phylogenetic distance of 0.05 nucleotide change per position. GenBank accession numbers are shown in brackets. Construction of the phylogenetic tree was performed by aligning sequences against the SILVA database (Pruesse et al., 2007) using the SINA online aligner (http://www.arb-silva.de/aligner/) and computing with the FastTree algorithm (Price et al., 2009).

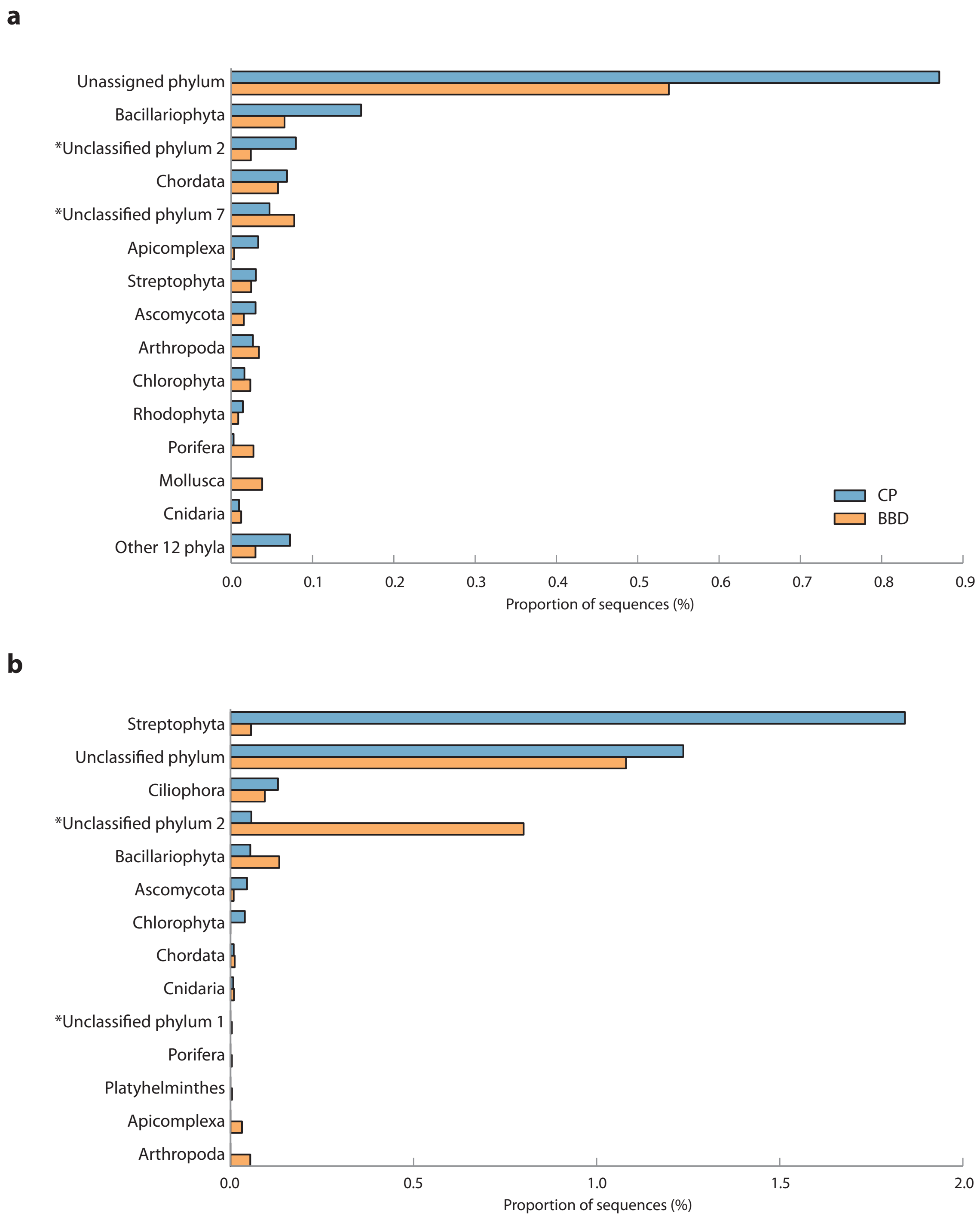

Supplementary Figure 6 Taxonomic compositions of eukaryotic annotated sequences in the metagenomes (**a**) and metatranscriptomes (**b**) derived from microbial lesions of cyanobacterial patches (CP) and black band disease (BBD) at the phylum level. All proportions are calculated as the relative abundance of sequences within the indicated taxa divided by the total number of sequences that were matched with Universally Conserved Protein sequences (E-value < 1.0E-5; asterisks denote taxa that have not been officially classified at the phylum level (see Supplementary Data 1 and 2)).

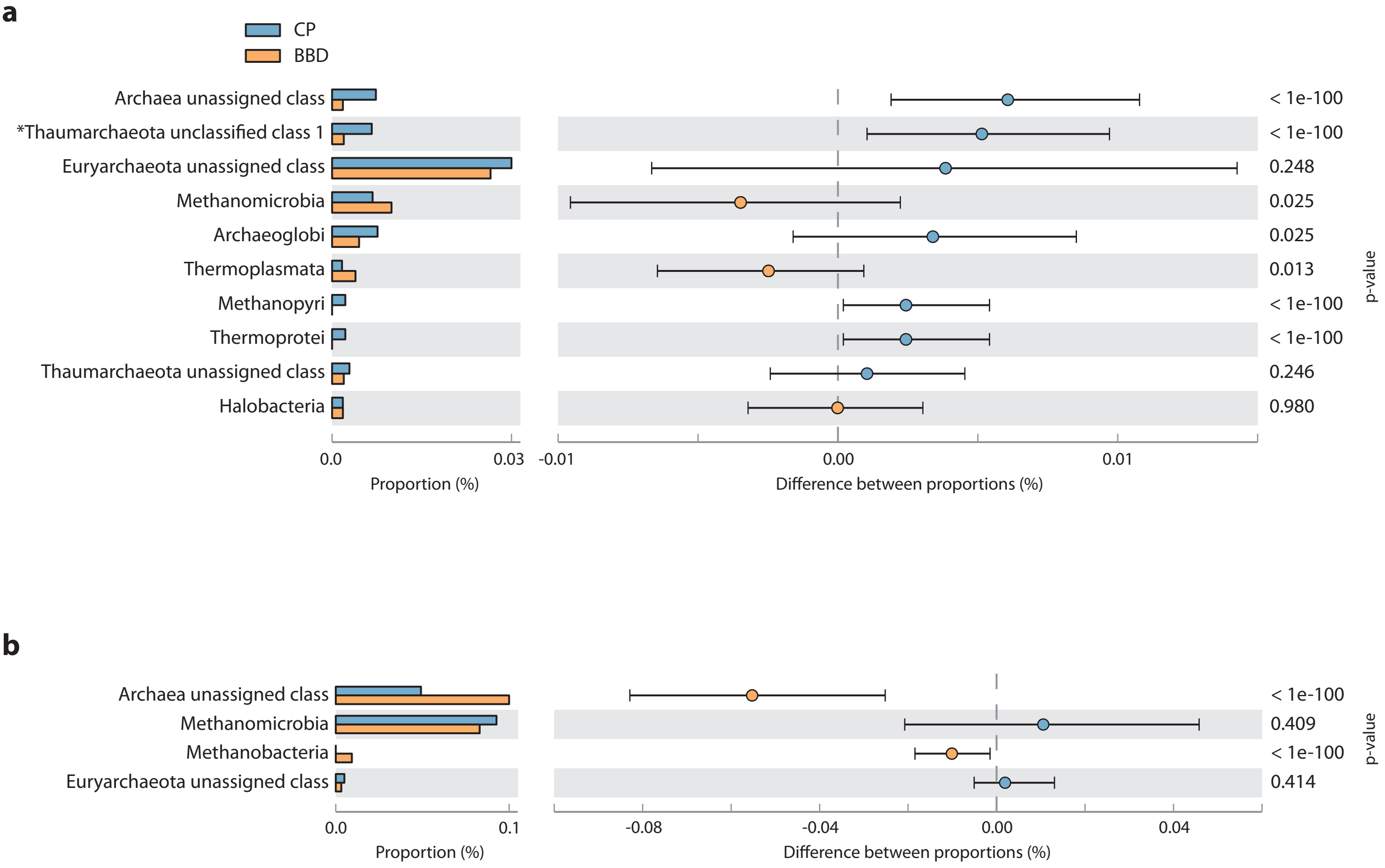

Supplementary Figure 7 Statistical comparisons of taxonomically annotated archaeal sequences at the class level in the metagenomes (**a**) and metatranscriptomes (**b**) derived from microbial lesions of cyanobacterial patches (CP) and black band disease (BBD). All proportions are calculated as the relative abundance of sequences within the indicated taxa divided by the total number of sequences that were matched with Universally Conserved Protein sequences (E-value < 1.0E-5; left, relative proportions; right, differences between proportions (negative value indicates BBD > CP) with 99.9% confidence intervals shown with error bars). Asterisk denotes a taxon that has not been officially classified at the phylum level (see Supplementary Data 1).

a

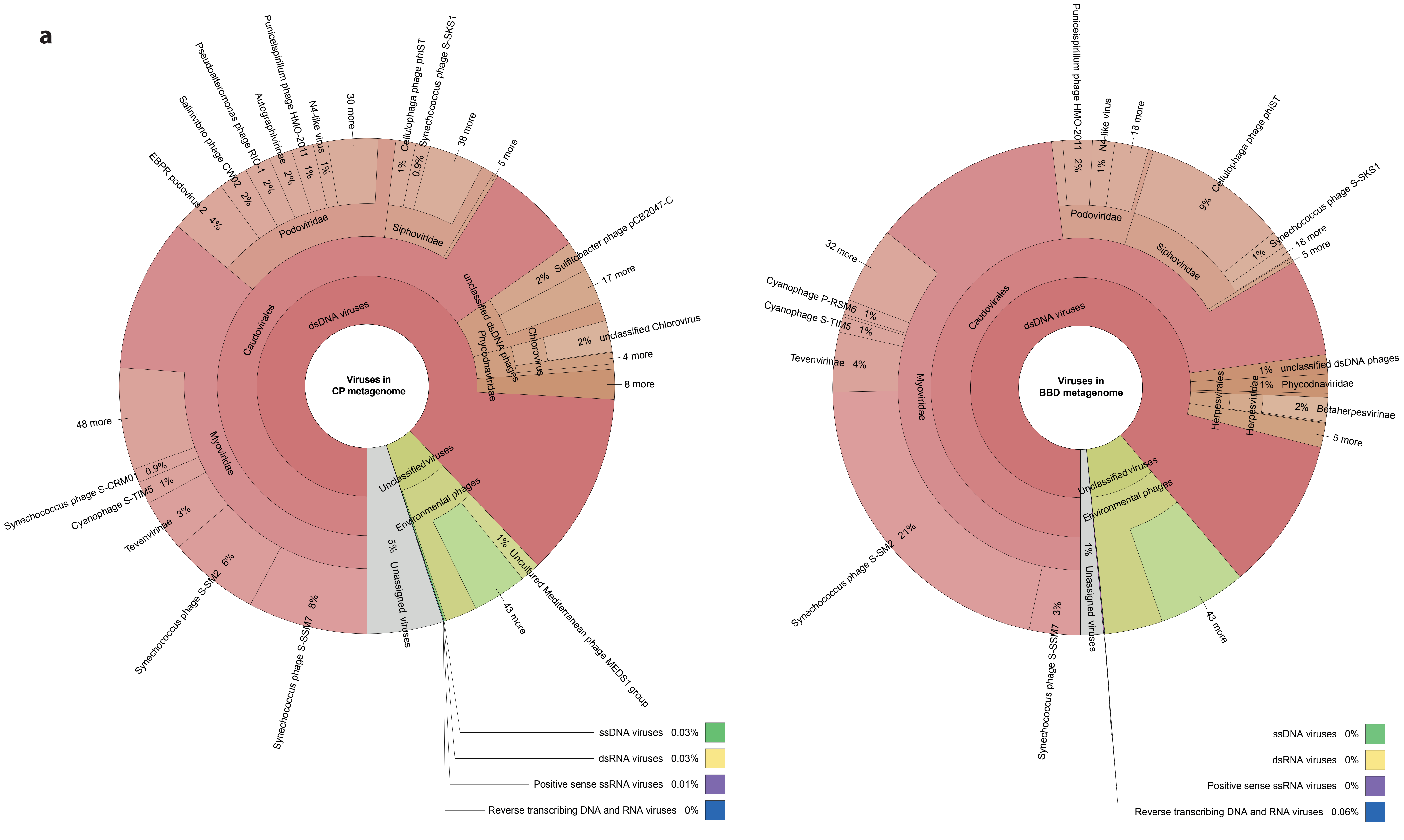

b

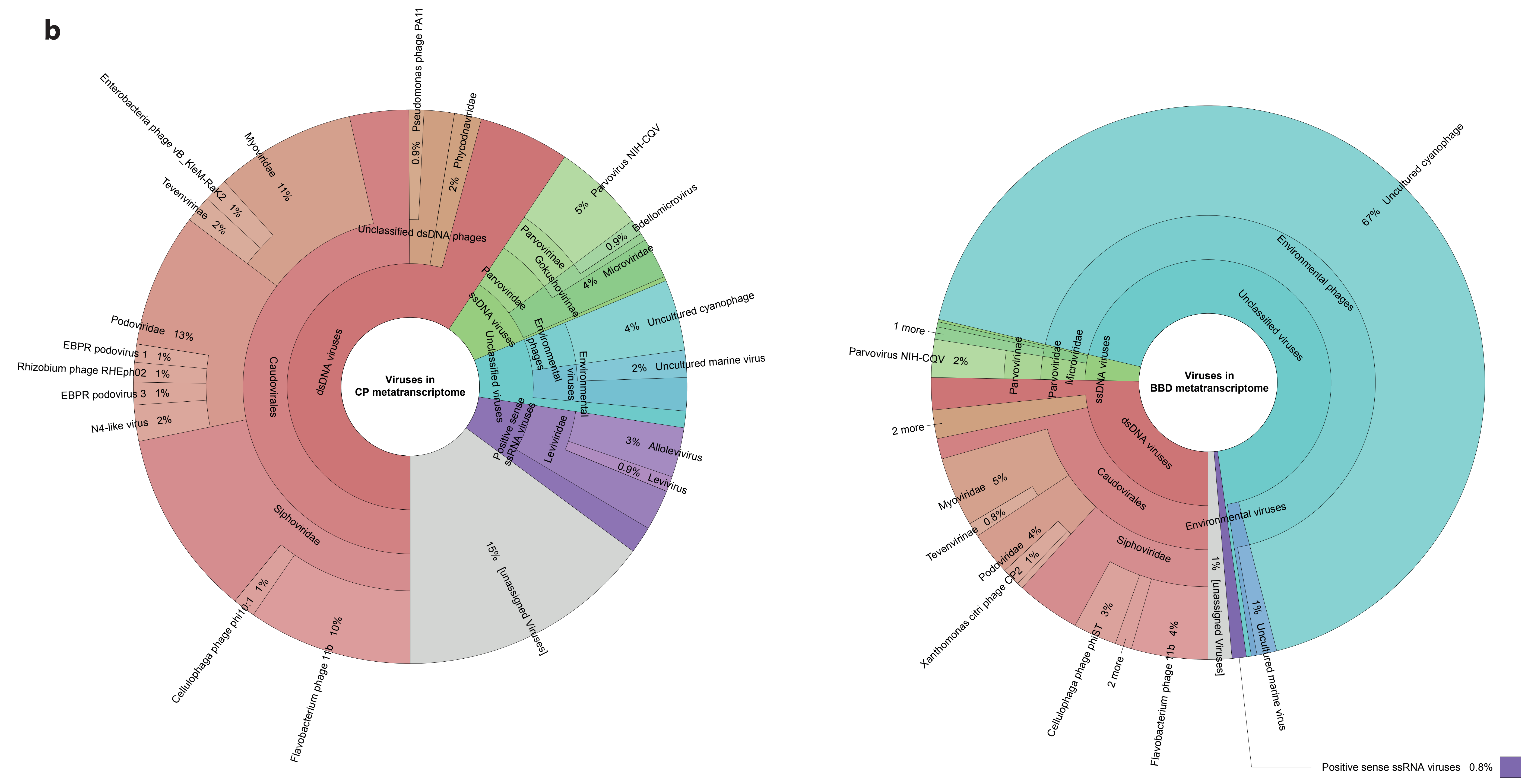

Supplementary Figure 8 Taxonomic compositions of viral annotated sequences in the metagenomes (**a**) and metatranscriptomes (**b**) derived from microbial lesions of cyanobacterial patches (CP) and black band disease (BBD) based on the sequence search against the NCBI-nr database (also see Supplementary Data 4 and 5).

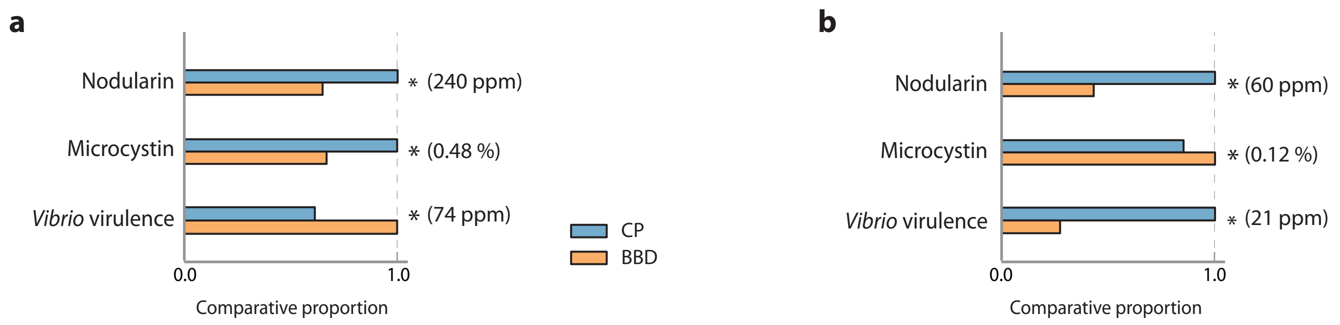

Supplementary Figure 9 Comparisons of the relative abundance of sequences affiliated with genes involved in the production of nodularin and microcystin and genes associated with Vibrio-associated virulence factors in the metagenomes (**a**) and metatranscriptomes (**b**) recovered from microbial lesions of cyanobacterial patches (CP) and black band disease (BBD). Graphs indicate relative proportions in comparison between CP and BBD, with whichever higher being set as 1.0. Actual proportions of the higher value in CP or BBD were indicated in brackets, calculated as the relative sequence abundance within all the sequences that were assigned to SEED Subsystems terms. Asterisks, $p < 0.001$; n.s., non-significant; ppm, parts per million.

Supplementary Table 1 Summary of the sampling scheme and resulting metagenomic and metatranscriptomic sequence libraries.

Sample ID*	Coral disease lesion*	Developmental stage (collection date)*	Extraction	Sequence library (pooled)	SRA accession ID
1	A	CP (2009-Oct-24)	DNA		
2	B	CP (2009-Oct-24)	DNA	CP metagenome	SRR3499156
3	C	CP (2009-Oct-16)	DNA		
4	A	BBD (2009-Nov-06)	DNA		
5	B	BBD (2009-Nov-06)	DNA	BBD metagenome	SRR3569370
6	C	BBD (2009-Oct-31)	DNA		
7	A	CP (2009-Oct-24)	RNA		
8	B	CP (2009-Oct-24)	RNA	CP metatranscriptome	SRR3569371
9	C	CP (2009-Oct-16)	RNA		
10	A	BBD (2009-Nov-06)	RNA		
11	B	BBD (2009-Nov-06)	RNA	BBD metatranscriptome	SRR3569372
12	C	BBD (2009-Oct-31)	RNA		

* Among different ‘Sample ID’s, ‘Coral disease lesion’ (A, B or C) corresponds to the identical lesion on the same coral colony, from which specimens were repeatedly collected at different dates (e.g. Samples 1, 4, 7 and 10). Where a combination of the ‘Coral disease lesion’ and ‘Developmental stage (collection date)’ is identical for different samples (e.g. Samples 1 and 7), it indicates that duplicate samples were collected from a single disease lesion on the same collection date. One of the duplicate samples was processed for DNA-extraction and the other was processed for RNA-extraction.

Supplementary Table 2 Summary statistics of metagenomic and metatranscriptomic sequence analysis.

	**Metagenome CP**	**Metagenome BBD**	**Metatranscriptome CP**	**Metatranscriptome BBD**
Raw sequences	72,705,148	79,412,006	89,843,846	140,380,726
Non-Ribosomal RNA sequences*	-	-	18,145,264	33,063,516
Taxonomically annotated sequences with UCP database	657,874	604,016	147,839	287,052
Matched sequences with NCBI-nr database	39,496,832	39,721,691	8,666,101	17,590,833
Functionally annotated sequences in SEED Subsystems	6,314,922	6,160,878	2,098,679	6,088,968

* Transcriptomic sequences other than ones coding structural ribosomal RNA (only calculated for metatranscriptomes). Screening of sequences coding structural ribosomal RNA (rRNA) from metatranscriptomes displayed that pre-sequencing subtraction of rRNA from total RNA extracts successfully enriched microbial functional transcriptomes (‘non-rRNA sequences’) above 20% in both CP and BBD metatranscriptomic sequences.

Supplementary Table 3 Metagenome-enabled transcriptomic analysis on the genomic bin Oce in black band disease (BBD) and cyanobacterial patches (CP). General functions (bold) and protein-coding assignments of genes that were differentially expressed in the genomic bin are shown with differences in rakings of the most expressed genes (positive values, BBD > CP; negative values, CP > BBD).

	BBD>CP	***Rank****		***CP>BBD***	***Rank****
**Translation**	30S ribosomal protein S5 (rpsE)	+177	**Stress response**	Protein RseC (rseC)	-742
	30S ribosomal protein S9 (rpsI)	+78		Transcriptional regulatory protein (cpxR)	-46
	50S ribosomal protein L7/L12 (rplL)	+52	**Oxidative stress resistance**	Alkyl hydroperoxide reductase subunit F (ahpF)	-153
	Elongation factor Tu (tuf1)	+40	**Virulence regulation**	3',5'-cyclic adenosine monophosphate phosphodiesterase (cpdA)	-174
**ATP synthesis**	ATP synthase subunit alpha (atpA)	+3252	**Polysaccharide synthesis**	RNA polymerase sigma-H factor (algU)	-462
	ATP synthase subunit alpha 2 (atpA2)	+130	**Cell lysis**	Membrane-bound lytic murein transglycosylase B (mltB)	-350
	ATP synthase, subunit I (LOR_35c02990)	+2901	**General secretion**	Type II secretion system protein E (xcpR)	-181
**Chemotaxis signaling**	Chemotaxis protein (cheW)	+2909	**Chemotaxis signaling**	Chemotaxis protein (cheW)	-344
	Protein phosphatase (cheZ)	+2739	**Fe-S cluster synthesis**	Protein SufA (sufA)	-256
**Fe-S cluster synthesis**	Cysteine desulfurase (iscS)	+1951	**Molecular redox**	Bifunctional NAD(P)H-hydrate repair enzyme (nnr)	-218
	HTH-type transcriptional regulator (iscR)	+725	**Protein folding**	Thiol:disulfide interchange protein (dsbC)	-406
**Fatty acid metabolism**	Isocitrate lyase (icl)	+2602	**Translation**	Histidine-tRNA ligase (hisS)	-312
**Glycerol metabolism**	Aerobic glycerol-3-phosphate dehydrogenase (glpD)	+2702			
**Virulence factor**	Extracytoplasmic function RNA polymerase sigma factor (sigL)	+2546			
**Cell motility**	Flagellin (r615_05030)	+176			
**Nucleoside metabolism**	Purine nucleoside phosphorylase (deoD)	+2413			
**Transcription**	DNA-directed RNA polymerase subunit alpha (rpoA)	+27			

* Rank difference between CP and BBD. Positive values indicate that the organism represented by the Oce bin had relatively higher proportions of transcriptomes mapped to the gene in BBD compared to CP, and negative values (-) indicate vice versa. Tables show predicted genes that are the most expressed (top 100 in either CP and/or BBD libraries) and their expressions are significantly different between CP and BBD ($p < 0.001$; bootstrap tests). Only genes assigned proteins with well-characterized specific functions are shown, and hypothetical genes and genes associated with unspecific function(s) were excluded.
